# Evaluation of co-speech gestures grounded in word-distributed representation

**DOI:** 10.3389/frobt.2024.1362463

**Published:** 2024-04-25

**Authors:** Kosuke Sasaki, Jumpei Nishikawa, Junya Morita

**Affiliations:** ^1^ Department of Informatics, Graduate School of Integrated Science and Technology, Shizuoka University, Shizuoka, Japan; ^2^ Department of Information Science and Technology, Graduate School of Science and Technology, Shizuoka University, Shizuoka, Japan; ^3^ Department of Behavior Informatics, Faculty of Informatics, Shizuoka University, Hamamatsu, Japan

**Keywords:** word-distributed representation, human-robot interaction (HRI), co-speech iconic gesture, natural language processing (NLP), robotics

## Abstract

The condition for artificial agents to possess perceivable intentions can be considered that they have resolved a form of the symbol grounding problem. Here, the symbol grounding is considered an achievement of the state where the language used by the agent is endowed with some quantitative meaning extracted from the physical world. To achieve this type of symbol grounding, we adopt a method for characterizing robot gestures with quantitative meaning calculated from word-distributed representations constructed from a large corpus of text. In this method, a “size image” of a word is generated by defining an axis (index) that discriminates the “size” of the word in the word-distributed vector space. The generated size images are converted into gestures generated by a physical artificial agent (robot). The robot’s gesture can be set to reflect either the size of the word in terms of the amount of movement or in terms of its posture. To examine the perception of communicative intention in the robot that performs the gestures generated as described above, the authors examine human ratings on “the naturalness” obtained through an online survey, yielding results that partially validate our proposed method. Based on the results, the authors argue for the possibility of developing advanced artifacts that achieve human-like symbolic grounding.

## 1 Introduction

In their daily life, people interact with a variety of artifacts. In doing so, they sometimes behave as if the objects have a kind of thinking ability (Nass et al., 1994; Nass and Moon, 2000). In this paper, the object causing such a behavior is called “an agent.” In other words, an agent is an artifact that can interact with people with its purpose, motivation, and intention (Levin et al., 2013; Kopp and Krämer, 2021; Human-Agent Interaction, 2023). We posit that understanding the factors that lead people to perceive artifacts as having these characteristics can facilitate enriching interactions, where humans naturally behave like in human-human interaction (HHI).

Research in the field of human-agent interaction (HAI) has explored factors causing people to perceive the agency in artifacts. Those factors are mainly classified into appearance (MacDorman and Ishiguro, 2006; Yee and Bailenson, 2007), behaviors (Heider and Simmel, 1944; Laban and Ullmann, 1971) and social contexts (Nass et al., 1994; Shiomi, 2023). Yet, all of them are categorized as external factors, omitting the discussion on the correspondences of internal states and processes (i.e., algorithms and representations (Marr, 1982)) between the artifacts and humans. We argue that studies focusing on the internal factors of these objects pave the way for a foundational design principle for agents. Such agents would appear to possess the aforementioned conditions of agency: purpose, motivation, and intention.

Building on this concept, the current study investigates the relationship between the perception of agency and addressing the symbol grounding problem (Harnad, 1990). Within our framework, symbol grounding is understood as an internal state in which the language (symbols) used by the agent is given some quantitative meaning extracted from the physical world. One possible form of assigning quantitative meaning to language appears in the co-speech gesture (body movement accompanied by verbal language). The correspondence between utterances and actions made by the agent allows humans to infer the existence of meaningful symbols in the agent.

In other words, we assume that some forms of gestures, which are implicitly generated in communicative situations, convey quantitative meaning attached to verbalized words. These gestures can be distinguished from culturally formed emblematic gestures. Rather than directly indexing a specific concept, the gestures focused here iconically enhance imagistic links between linguistic form and meaning (McNeill, 1992; Murgiano et al., 2021). Murgiano et al. (2021) also claimed that such an iconic gesture is part of multimodal systems conveying imagistic meaning in communicative contexts. Thus, a similar role is observed in the prosody that accompanies spoken words. Herold et al. (2011) reported that children deduce novel meaning of antonyms (e.g., “small” vs. “big”) by leveraging prosodic features such as intensity (i.e., a loud and slow voice is connected to a big object).

The connection between a physical image and word meaning (the degree of symbol grounding) varies with word categories. Concrete categories naturally exhibit stronger links than abstract concepts (Utsumi, 2020). Nonetheless, abstract words can also possess imagistic meaning. Lakoff and Johnson (1980) discussed how language is metaphorically shaped through schemata that involve movement within the external world. This concept, known as an image schema, allows for the preservation of the external world’s imagery while linking through metaphorical expressions. For instance, when a speaker utters the phrase, “I have an important idea,” we can envision a scenario where the speaker’s hand gesture expands to signify the idea’s perceived significance. In this gesture, the magnitude of the concept is metaphorically represented through the spatial dimensions defined by the speaker’s body structure. Such a set of metaphors connecting a physical experience and an abstract concept is known as a primary metaphor (Grady, 1997).

Existence of the mechanism of exchange for these representations (symbols and quantities) is also supported by various theories in the field of cognitive science. According to the reference frames theory by Hawkins (2021), a continuous space exists behind each concept, mediating language use. Similarly, Tversky (2019) claimed that human language and thoughts originally come from physical experience made in a continuous time and space. In her discussion, the meanings of words are essentially embedded in our living physical world. Other similar discussions are also found in literature in the field of cognitive linguistics (Pinker, 2007).

Summarizing the above background of cognitive science, the authors consider that the gestures generated from a mechanism that is analogous to what humans hold can lead to a realization of the “intrinsically naturalistic” interactions with the agent, where people can perceive “communicative intention (Grice, 1989)” in the agents. In order to construct such a mechanism, a model of word meaning is important. As already noted by the above theories (Pinker, 2007; Tversky, 2019; Hawkins, 2021), word meanings are not defined discretely or independently, but are considered to be defined in a continuous space in which words are interconnected. In the history of natural language processing, statistical analyses (bag of words, co-occurrence frequency, or principal component analysis from word vectors) have been applied to corpora derived from human language operations to capture the semantic relations between words. More recently, vector representations (word-distributed representations) collapsed into the middle layer of a neural network (Bengio et al., 2000) have become the mainstream method for understanding words’ quantitative meanings. Such an approach is still evolving and has led to the construction of a variety of large-scale language models (LLM) that enable HAI with human natural communication media (natural language) (Brown et al., 2020; Chowdhery et al., 2023; OpenAI, 2023).

Various quantitative images such as “size” and “speed” can be assumed in the space where words are positioned (Grand et al., 2022). Among those, we focus on the “size images” as a first step to obtain quantitative representations of words related to physical image embedded in the space. Since this image has been frequently utilized in numerous studies (Grady, 1997; Herold et al., 2011), it is suggested that the most representative image for our investigation. By creating iconic gestures (physical images) for a robot through the conversion of “size images” into physical representations, we aim to develop an agent that achieves symbol grounding, which leads to actions that reflect the quantitative images of words. Our objective is to determine whether such embodiment in an agent (robot) leads to an increase in human perception of agency.

More specifically, as an initial step toward the above objective, we set the following research questions:

1 How can “size images” evoking an agency perception be extracted from the vector space of word-distributed representations?
2 What forms of gesture expression are effective in constructing more natural interaction based on the agency perception?

To address the first question, we introduce a method for extracting “size images” using word-distributed representations and evaluate this method through two experiments. These experiments employ different approaches for associating “size images” with “physical images” in a robot. By comparing the outcomes of these experiments, we aim to investigate the second question. Before presenting the experiments, we introduce a technological background leading to the method of this study and the method of generating “size images” in the following sections.

## 2 Related works

As a background of our method, we introduce research on modeling word meaning and research on gesture generation for robots and agents. Based on the review of those related works, we outline our specific approach to gesture generation.

### 2.1 Modeling word meaning

The meaning of a word or concept can be modeled by several approaches. One traditional approach is to write down the meanings of concepts circulating in society manually. Large-scale databases such as WordNet (Miller, 1995) and ConceptNet (Speer et al., 2017) have been developed so far. These databases define the normative knowledge structure in society.

On the other hand, in recent years, there have been many approaches to statistically capture the meaning of concepts based on the way people use language in their daily lives. The word-distributed representation (Bengio et al., 2000) considers a word as a “pointer” embedded in a vector space. In this framework, the meaning of a word is regarded as the relationship (distance or similarity) between words in the vector space. The underlying idea here is the distributional hypothesis that “words which are similar in meaning occur in similar contexts (Rubenstein and Goodenough, 1965; Sahlgren, 2008)”.

Attempts have been made to extract words’ quantitative images by using word-distributed representations. For example, Utsumi (2020) used word-distributed representations to classify words into attributes and compared them with the classifications obtained from human data. The results suggests that the vector space of word-distributed representation captures aspects of human knowledge, showing that abstract concepts are more deeply (remotely) embedded in word distributions than in words with physical meanings associated with animates.

In addition, Grand et al. (2022) proposed a method for extracting context-dependent relations using word-distributed representations. Context-dependent relations imply that a word like “dog” can embody multiple semantic features such as “size,” “intelligence,” and “danger,” with a particular feature becoming prominent depending on the context. This study shows that by projecting word vectors onto an axis representing a focused feature, it is possible to simulate human estimation of the quantitative features in various objects. Thus, it is suggested that human quantitative images of words are embedded in word-distributed representations. In other words, the quantitative meanings of concepts that humans have physically acquired are inherent in word-distributed representations created from our daily language use.

### 2.2 Gesture generations in human-agent interaction

The current study focuses on symbol grounding as a factor inducing agency perception. Regarding this focus, Section 1 introduced studies showing the relation between symbol grounding and multimodal communication (Murgiano et al., 2021).

In the context of HAI studies, multimodal interaction has also been extensively examined. Among these, human gestures have been treated as a main modality that significantly influences verbal communication (Maricchiolo et al., 2020). To approach this, data-driven methods that learn from human gestures by using machine learning techniques such as deep learning have become popular. For example, Saund et al. (2022) analyzed the relationship between body movement and meaning to generate effective gestures by virtual agent.

There have also been many studies on gesture generation from multimodal language corpus. Lin and Amer (2018) use Generative Adversarial Networks (GAN) to control joints by mapping embedded words to the space of body movements. Ahuja and Morency (2019) also proposed a method called “language2pose” that integrates language and body movements through end-to-end learning. More recently, Tevet et al. (2022) used a diffusion model to generate human body movements and use sentences and actions as input. Their study confirmed that gestures generated from both inputs were evaluated better than gestures generated by other generation models. Furthermore, possibility of natural gesture generation or selection is explored by using LLM (Brown et al., 2020; Chowdhery et al., 2023; OpenAI, 2023), which has become popular in recent years. Hensel et al. (2023) have shown that LLM can be used to select hand gestures that are compatible with the content of speech. Yoshida et al. (2023) have successfully generated emblematic gestures by incorporating LLMs into humanoid robot motion generation.

In the context of co-speech gesture generation, Yoon et al. (2019) applied deep learning technology to generate various gestures, including iconic, metaphoric, deictic, and beat gestures. Ishii et al. (2018) also proposed a model of co-speech gesture focusing on appropriate timing. In their study, Conditional Random Fields (CRFs) were used to parse information from natural language.

### 2.3 Top-down gesture from abstract index

As described thus far, numerous studies have focused on bottom-up approaches that extensively learn low-level features from human motion data. Although these approaches have proven effective for generating natural gestures, using the bottom-up approach to identify intrinsic yet infrequently occurring features related to spoken words remains challenging due to the inherent bias of deep learning technologies towards the majority of data samples. Therefore, a top-down approach that targets specific aspects of word meanings is necessary to achieve a robot that acts as an agent “grounded to the external world,” as described in Section 1.

**FIGURE 1:**
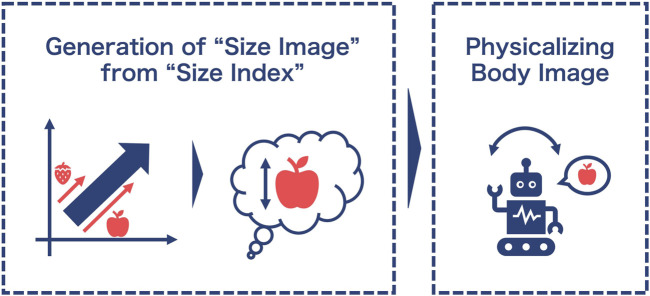
Gesture generation proposed by Sasaki et al. (2023).

Based on these ideas, Sasaki et al. (2023) proposed a gesture generation method by extracting intrinsic images of words as shown in Figure 1. The details will be explained in the next section, but their method has advantages in assuming an abstract axis (index) to be extracted from the data, by following Grand et al. (2022). We consider that such an intentional setting of an axis is essential to represent communicative intention in the agent (Grice, 1989). However, their study did not present sufficient evaluations of the variety of body expressions. Therefore, this study extends the previous method (Sasaki et al., 2023) to evaluate the effects of physical images generated from the abstract index on agency perception. In the following sections, we describe the method of constructing “size index” and “size images,” which are the process presented in the left side of Figure 1.

## 3 Generating “size images” of words

In this study, we generate gestures of embodied agents (robots) by using the method proposed by Sasaki et al. (2023). After presenting an overview of the method, we apply it to survey data to extract the “size images” of words.

### 3.1 Basic method

**FIGURE 2:**
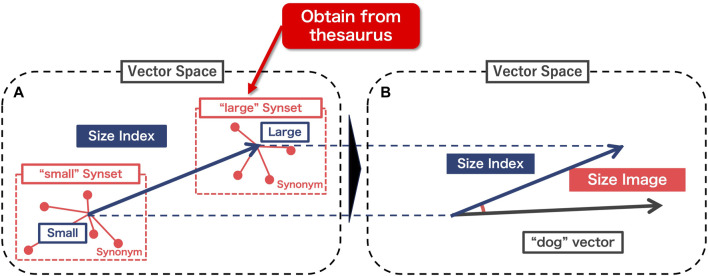
Procedure of the proposed method. **(A)** Construction of “size index”, **(B)** Calculation of “size image”.

Figure 2 illustrates the methodology employed in this study to generate “size images.” Figure 2A details the procedure for extracting a “size index” applicable to any word in the word-distributed representation. This involves identifying quantitative dimensions related to the attribute of size. Following this, Figure 2B outlines the process of creating a “size image” for a specific word using the derived size index. The output of this step is utilized to translate the abstract semantic feature into a physical representation (robot movement) that can be recognized within the context of human-agent interaction, thereby facilitating the symbol grounding of the word based on its size attribute.

These processes adapt and modify the approach presented by Grand et al. (2022), which was introduced in Section 1. To overcome the limitations of Grand et al.’s method, which we will discuss later, our method (Sasaki et al., 2023) employs an approach that combines word-distributed representations with a human-curated thesaurus, introduced in the beginning of Section 2.1. This approach reduces the arbitrariness of the method and enhances its applicability to languages with comprehensive linguistic resources. In this study, to assess the method’s applicability beyond English, we utilize a linguistic resource developed for the Japanese language.

The remainder of this subsection details the specific procedure employed in this study for extracting the “size index” and constructing the “size image”.

#### 3.1.1 Composition of “size index”

We first present Grand et al. (2022)’s method of extracting the “size index” (the blue arrow in Figure 2A). In this method, an axis with a meaning specific to “large,” is constructed by subtracting a polar vector with a meaning of “small” (Small in Figure 2A, the antonym of “large”) from a different polar vector with a meaning of “large” (Large in Figure 2A). To define the poles, we only need to extract the coordinates of the “large” and “small” values in the distributed multidimensional vector space. However, in addition to their size-related meanings, these two words have extra meanings that derive from their adjectival roles in the sentence^1^. To exclude such meanings unrelated to the degree of “size,” Grand et al. (2022) defined a set of synonyms (red dotted square in Figure 2A) that have the same role as “large” and “small” in the distribution word representation. Then, the polar coordinates are determined by computing the mean vector of these synonyms, respectively.

Thus, the “size index” ***I*** is defined by the following equation:

$$I=\frac{\sum_{i=1}^{n}l_{i}}{n}-\frac{\sum_{j=1}^{m}s_{j}}{m}$$

where ***l***_***i***_ and ***s***_***j***_ are word belonging to the set of “large” synonym vectors (*Synset*_*L*_ = {***l***_**1**_, ***l***_**2**_, … , ***l***_***n***_}) and the set of “small” synonym vectors (*Synset*_*S*_ = {***s***_**1**_, ***s***_**2**_, … , ***s***_***m***_}).

#### 3.1.2 Composition of “size image”

Once the “size index” is defined, we can calculate “size image” from the index. Figure 2B shows the calculation of the “size images” as the cosine similarity between the “size index” and the input word vector. Thus, the size image *S* is calculated by

$$S=\frac{I\cdot w}{\left\|I\|\;\|w\right\|}$$

where ***w*** represents the input word vector. In this method, the larger this value is, the larger the word is assumed.

#### 3.1.3 Selection of synset

The limitation of Grand et al.’s method is arbitrariness in selecting *Synset*_*L*_ and *Synset*_*S*_. In their study, *Synset*_*S*_ consisted of “tiny” and “little” while *Synset*_*L*_ was constructed by “big” and “huge.” Nevertheless, they didn’t specify the criteria for selecting these synonyms.

To address this issue, and to compose a “size index” properly matching human perception, it is important to set up a set of synonyms for the polar words (“large” and “small”) without any arbitrariness. A possible method is leveraging a standardized thesaurus. However, a thesaurus does not automatically determine the appropriate synonym set. Words are usually polysemous and have multiple meanings. In a thesaurus, a set of synonyms for a word is defined as a synset for each meaning. To improve the consistency with human perception, it is necessary to select appropriate synsets. In this study, we seek the combination of synsets that maximizes the distance between “large” and “small” words obtained from human reports to determine the polar coordinates consistent with human perception. In this procedure, we first prepare *Synset*_*L*_ associated with the word “large” and *Synset*_*S*_ associated with the word “small”.

We also prepare a set of words *Large*_*h*_ that humans perceive as “large” and a set of words *Small*_*h*_ that humans perceive as “small.” In extracting *Large*_*h*_ and *Small*_*h*_, it is necessary to distinguish categories to which the word refers. According to Tversky (2019) and others, the meaning of a concept is originally composed of human movement. However, as shown by Utsumi (2020), physical quantities are not expected to be strongly embedded in word-distributed representations composed of socially published documents. Either way, the scale of the “size index” has the possibility to be changed by the categories the word belongs to. Following such discussions, this study assumes.

• *Large*_*h*, *animate*_
• *Large*_*h*, *inanimate*_
• *Large*_*h*, *intangible*_

as subclasses of *Large*_*h*_, and.

• *Small*_*h*, *animate*_
• *Small*_*h*, *inanimate*_
• *Small*_*h*, *intangible*_

as subclasses of *Small*_*h*_.

In the selection process of the synset combinations, the “size index” ***I***_***i,j***_ is composed for *Synset*_*S*,*i*_ (∈ *AllSynseet*_*S*_) and *Synset*_*L*,*j*_ (∈ *AllSynseet*_*L*_). The “size images” of the human-perceived small word ***w***_***S,k***_ (∈ *Small*_*h*_) and large word ***w***_***L,l***_ (∈ *Large*_*h*_) are severed to calculate the “size images” *S*(***I***_***i,j***_, ***w***_***S,k***_) and *S*(***I***_***i,j***_, ***w***_***S,l***_), respectively. Those individual “size images” are aggregated into average images as *S*(***I***_***i,j***_, ***w***_***L***_), and *S*(***I***_***i,j***_, ***w***_***S***_). From those indices, the “size index” of the combination of *Synset*_*L*_ and *Synset*_*S*_ that maximizes the difference (*S*(***I***_***i,j***_, ***w***_***L***_) − *S*(***I***_***i,j***_, ***w***_***S***_)) are selected as the optimal index to compose human compatible “size images”.

### 3.2 Application of the method

The construction of the “size index” described so far requires a vector representation of words (***w***), a thesaurus (*AllSynset*_*S*_, *AllSynset*_*L*_), and human perceived small/large words (*Small*_*h*_, *Large*_*h*_).

Of these, this study uses the Japanese Wikipedia entity vector developed by (Suzuki et al., 2016), which we call JWikiEntVec in this paper, as a distributed representation model to construct a vector of words (***w***). This is a trained model built by word2vec (Mikolov et al., 2013). We used this because the model is well used in Japanese academic societies^2^. Also the development method and the data set used to construct the model are clearly presented by the authors of this model. These characteristics make it particularly advantageous for foundational research like this study, despite the model’s performance not being as high as that of more advanced LLMs.

Furthermore, we employed the Japanese WordNet (Bond et al., 2009) for the selection of synonyms. This thesaurus contains 28 synsets for “large” and 14 synsets for “small.” Words not included in JWikiEntVec and synsets with no synonyms were excluded from the later analysis. As a result, we obtained 23 synsets for “large” and 13 synsets for “small.” The “size index” was calculated for the combinations of these synsets (23 × 13 = 299).

The human word sets (*Small*_*h*_ and *Large*_*h*_) were collected through a questionnaire survey whose participants (*n* = 100) were recruited from a Japanese crowdsourcing site (Lancers). The participants were asked to write down five “large” and “small” words for animate, inanimate, and intangible concepts (30 words in total). Table 1 shows the top five words and their frequency for each question. From the table, we find the word “Mind” appears in the top five words for both large and small intangible. This duplication is considered to indicate the ambiguous nature of the meaning of this word. Therefore, this study used “Mind” in both *Small*_*h*_ and *Large*_*h*_ and calculated the “size index” by using these 29 words as the elements of *Small*_*h*_ and *Large*_*h*_.

**TABLE 1:** Responses obtained for each question (Top five words).

Animate			
Large		Small	
Word	Freq	Word	Freq
Elephant (zo-u)	85	Ant (a-ri)	74
Whale (ku-ji-ra)	68	Daphnia (mi-ji-n-ko)	33
Giraffe (ki-ri-n)	60	Mosquito (ka)	31
Bear (ku-ma)	25	Tick (da-ni)	30
Hippopotamus (ka-ba)	19	Fleas (no-mi)	23

Inanimate			
Large		Small	
Word	Freq	Word	Freq
Tokyo Sky Tree (to-kyo-su-ka-i-tu-ri)	45	Sand (su-na)	26
Mt. Fuji (fu-ji-sa-n)	35	Beads (bi-zu)	20
Tokyo Tower (to-kyo-ta-wa)	29	Needle (ha-ri)	26
Everest (e-be-re-su-to)	22	Microchip (ma-i-ku-ro-chi-ppu)	15
Pyramid (pi-ra-mi-ddo)	18	Screw (ne-ji)	14

Intangible			
Large		Small	
Word	Freq	Word	Freq
Space (u-tyu-u)	36	Mind (ko-ko-ro)	13
Love (a-i)	18	Jealousy (si-tto)	9
Dream (yu-me)	17	Envy (ne-ta-mi)	7
Mind (ko-ko-ro)	16	Vanity (mi-e)	6
Sea (u-mi)	15	Point (te-n)	5

*Words are translated from Japanese. Japanese pronunciation in hepburn romanization is presented in parentheses.

**FIGURE 3:**
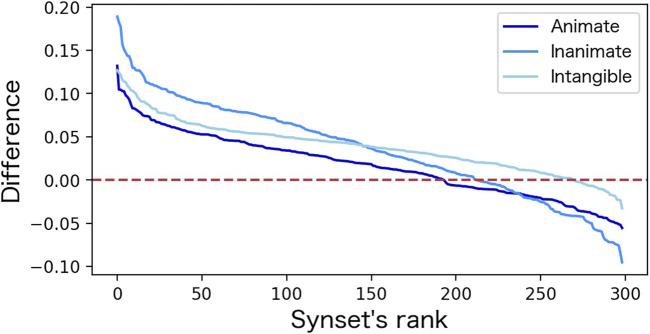
Distribution of “size image” difference.

Figure 3 shows the differences (*S*(***I***_***i,j***_, ***w***_***L***_) − *S*(***I***_***i,j***_, ***w***_***S***_)) calculated for the 299 combination of synsets. The horizontal axis of this figure corresponds to the combination of synsets ordered by rank. Overall, there are many combinations where the difference of the “size image” is larger than 0 (above the red dotted line), indicating that the size index calculated for more than half of the synset combinations is consistent with the human image.

Table 2 shows the highest-ranked synset combinations (with the largest difference), the lowest-ranked synset combinations (with the smallest difference), and the synonyms in each synset. The combination of the synset with the largest difference is “larger-than-life” and “peanut,” and the combination of the synset with the smallest difference is “major” and “small-scale”.

**TABLE 2:** Top and bottom synsets combinations.

	Top synset		Bottom synset	
word	“large”	“small”	“large”	“small”
synset	larger-than-life	peanut	major	small-scale
meaning	very impressive	unimportant	effective	very small
Synonym 1	magnificent (so-da-i)	insignificant (bi-bi-ta-ru)	great (o-o-ki-na)	modest (sa-sa-ya-ka)
Synonym 2	large scale (da-i-ki-bo)	only (wa-zu-ka)	significant (o-o-ha-ba)	cottage (re-i-sa-i)
Synonym 3		trivial (sa-sa-i)	serious (ju-da-i)	tiny (ti-ttya-i)
Synonym 4		cheap (ya-su-ppo-i)		
Synonym 5		slight (ke-i-bi)		

*Words and meanings are translated from Japanese. Japanese pronunciation in hepburn romanization is presented in parentheses.

## 4 Experiment 1: “size image” in effort

The following two sections present an evaluation of the physical images (iconic gestures) generated from the “size images” composed in the previous section.

To address the first research question presented in Section 1, we tested the above procedure of selecting synsets. Thus, the gesture generation using the top-ranked synset combination and that using the bottom-ranked synset combination are treated as the *proposed* and *controlled* methods, respectively. If the procedure described in the previous section successfully extracted the axis that grounds the symbol to the physical world, and if the correspondence between the axis and the body is compatible with what people own, we can claim that the proposed method is effective to extract “size image” that evokes agency perception from the vector space of word-distributed representation.

Regarding the second question, we can consider several possible methods for mapping the “size image” to “physical image.” Dance theories generally pursue a physical expression that effectively externalizes the human internal states. Among these theories, Laban movement analysis (Laban and Ullmann, 1971) has been widely used in the field of HAI (Ishino et al., 2018). This theory assumes two modalities in the correspondence between human internal states and body: *shape* for posture and effort for movement. In Experiment 1, we mapped the axis of space, which is one of the axes of the effort modality to the “size index.” In other words, the amount of movement is considered to be larger when a large concept is recalled. In the following section, we explain the construction of body movements based on this idea.

### 4.1 Methods

#### 4.1.1 Materials

The procedure of generating physical image (iconic gesture) according to the “size image” is shown below.

1 Setting large and small movements. Mapping the “size image” to the posture composed of the body parts. For this purpose, we define the body posture corresponding to the smallest and largest words recognized by humans. Using this posture as a reference (0 for the image of the smallest word and 1 for the image of the largest word), the “size image” of each word is positioned in the range from 0 to 1.
2 Calculation of parameters at each joint. The above scaling is applied to the angles of each joint that constitute the posture.
3 Generation of physical image. A gesture is generated based on the values obtained by step 2. This generation is assumed to be made simultaneously with the utterance of the word.

In order to embody the above steps, we used Sota, a small communication robot by Vstone^3^. Sota’s body movements are controlled by nine joints (one torso, three necks, two shoulders, and two arms joints). By controlling the angle and speed of these joints, Sota can generate a variety of movements. In addition, Sota has a speech function and can speak any word while simultaneously displaying gestures.

**FIGURE 4:**
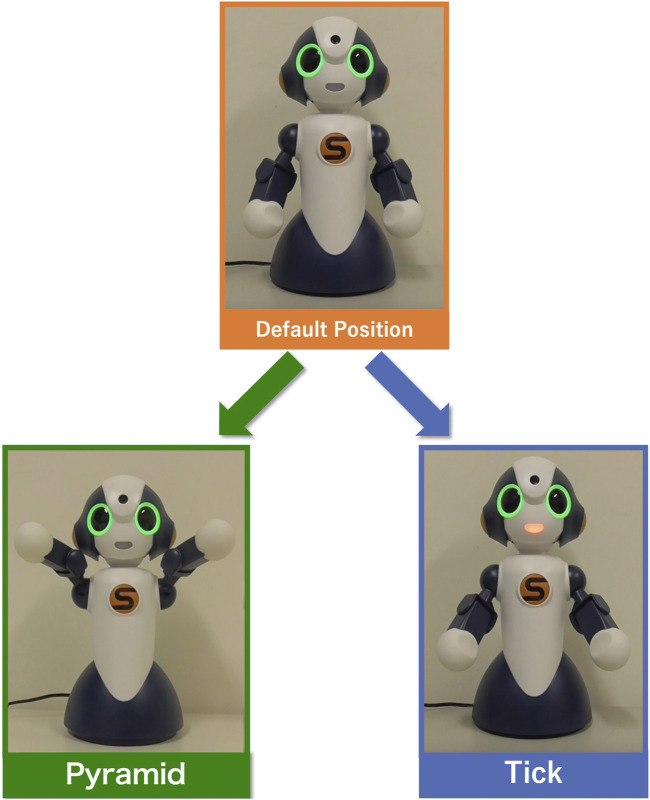
Examples of Sota gestures (left: “Pyramid”, right: “tick”).

In this study, the parameters of Sota’s arm and shoulder joints were instantiated by “size image” of each word. Sota’s default posture in this study is shown in the upper image in Figure 4, with its shoulders down and arms slightly bent. From this state, the parameters of the arm and shoulder joints are changed to generate a gesture corresponding to the size of the word. Table 3 shows the maximum and minimum values of the “size image”, as well as the parameters of the shoulder and arm joints and the default position that corresponds to them. The “size image” computed for both the proposed and controlled methods is mapped to these values: 0 for the minimum and 1 for the maximum in the proposed method, with the reverse applied in the control method.

**TABLE 3:** Maximum and minimum values of parameters for “size image” and each joint in Experiment 1.

	Maximum	Minimum	Default
**Size Image (proposed)**	0.39	−0.17	-
**Size Image (control)**	0.28	−0.12	-
**Shoulder angle**	75	−68	−70
**Arm angle**	−20	88	90

**TABLE 4:** Maximum and minimum values of parameters for “size image” and each joint in Experiment 2.

	Maximum	Minimum	Default
**Size Image(top synset)**	0.39	−0.17	-
**Size Image(bottom synset)**	0.28	−0.12	-
**Shoulder angle**	30	−30	−70
**Arm angle**	−20	90	20
**Neck angle**	10	−10	0

#### 4.1.2 Design and measures

In the experiment, the physical images generated by the above procedure were recorded as movies. Supplementary Material included examples of the movies (each for about 4 s duration^4^) and all the pictures showing poses representing each word, captured from the end of each movie. In this experiment, 30 words (with one duplication) in Table 1 were used to generate physical images for both the control and proposed methods^5^. Among them, two examples are shown in the lower images of Figure 4. The lower left and right images depict gestures for the words “tick (da-ni)” (the smallest animate concept) and “pyramid (pi-ra-mi-ddo)” (the largest inanimate concept), respectively. Thus, a small “size image” results in the robot making small movements from the default position^6^, while a large “size image” results in larger movements.

The participants were asked to observe those movies and rate the naturalness of the correspondence between the robot’s movements and the words it speaks on a 5-point scale (1: not at all natural—5: very natural). The standard of “naturalness” here assumes a natural communication between humans (Kopp and Krämer, 2021). In human communication, people usually try to achieve, intention sharing (i.e., mutual understanding) (Tomasello, 2010). Therefore, we specifically asked the participants to rate whether or not they feel that the robot understands the meaning of the words as humans would. As the question indicates, this rating demands the participants to perceive the robot’s internal state from the short movie. By presenting such a question, evaluating the agency perception in terms of communicative intention (Grice, 1989) would be possible.

#### 4.1.3 Participants and procedure

The 300 participants recruited from Lancers joined the experiment after reading the instructions provided on the request screen (reward: 110 JPY). The instructions explained the evaluation procedure, the definition of naturalness, and the obligation to answer dummy questions. After agreeing to the above instructions, participants were presented 14 movies, which were randomly selected for each participant from 58 movies (2 conditions × 29 words in Table 1). In between the evaluation of the movies, a dummy question in which the participants were asked to answer a specified number was inserted.

### 4.2 Results

**FIGURE 5:**
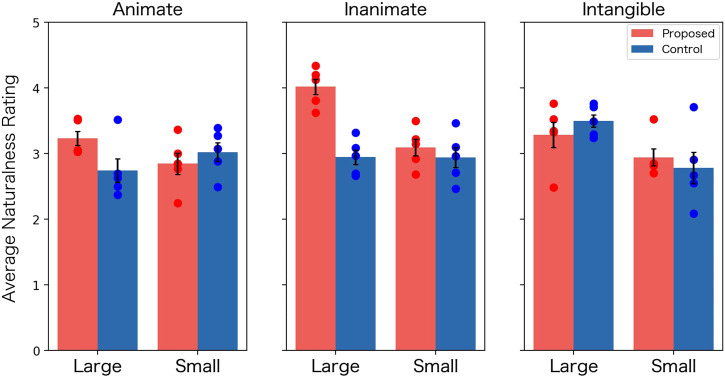
Mean rating of naturalness in Experiment 1 (Error bars: standard errors, Dots: ratings for naturalness for each word).

Nine participants who answered the dummy questions incorrectly were excluded from the following analysis. From the remaining 291 participants’ responses, the average of the ratings was calculated for the 29 words in each condition. Utilizing this value as a unit of analysis, we calculated the average of 12 naturalness ratings (condition × size × category)^7^, as shown in Figure 5. By using these values, we try to test the following hypotheses:

1 The proposed method has a greater effect on the ratings than the control method.
2 The above effect is affected by the difference between the word categories.

The first hypothesis directly relates to the first research question introduced in Section 1. The second hypothesis was explored in response to previous studies (Utsumi, 2020), which have demonstrated that the impact of physical experience on word meaning diminishes in abstract concepts. Consequently, it is advantageous for future research to elucidate the extent to which symbol grounding influences the perception of agency.

We conducted a three-way [condition (proposed vs. control) × size (large vs. small) × category (animate vs. inanimate vs. intangible)] analysis of variance (ANOVA) to test the above hypothesis. Among the effects obtained from the ANOVA, we focused on the main effect of the condition and the interactions involving the condition and the category (a second-order interaction between the condition, the size and the categories, and a first-order interaction between the condition and the category). During this process, the significance level was set to 0.10, reflecting small sample size in this study (*n* = 5 for each condition). We also control type-1 error by reporting corrected *p*-values as q-value calculated using the Benjamini-Hochberg (B-H) method.

From the analysis, we obtained a significant main effect of the condition (*F* (1, 48) = 6.48, *p* = 0.01, *q* = 0.04, *η* ^2^ = 0.07), confirming that the proposed condition was evaluated more naturally than the control condition. However, we also found a significant second-order interaction between the condition, the size, and the categories (*F* (2, 48) = 4.09, *p* = 0.02, *q* = 0.05, *η* ^2^ = 0.14) and a significant interaction between the condition and the categories (*F* (2, 48) = 3.78, *p* = 0.02, *q* = 0.05, *η* ^2^ = 0.08), suggesting that the difference in naturalness between conditions depends on other factors.

To examine the details of the interaction effect, we conducted *post hoc* two-way [condition (proposed vs. control) × size (large vs. small)] ANOVAs for each category. The *p*-values were corrected using the BH-method, accounting for nine tests in total (one interaction and two main effects for three ANOVAs). Significant effects (*p* < .05) related to the condition were observed only in the inanimate category (the main effects of condition: *F* (1, 16) = 18.72, *p* < .01, *q* < .01, *η* ^2^ = 0.53 and the interaction: *F* (1, 16) = 10.50, *p* < .01, *q* = 0.02, *η* ^2^ = 0.66). The simple main effect of the condition in the inanimate category was observed for the large concept (*F* (1, 16) = 21.29, *p* = 0.01, *q* < .01, *η* ^2^ = 1.78). These results suggest that the proposed condition was evaluated as significantly more natural than the control condition for the large inanimate concept.

**FIGURE 6:**
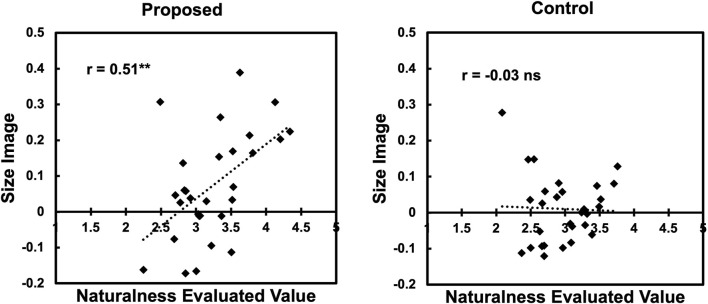
Correlation between naturalness and “size image” in Experiment 1.

The above results partially align with the previous study (Utsumi, 2020). Additionally, the result revealed an effect of the “size” (small vs. large) on agency perception, which was not expected. To investigate this effect further, a *post hoc* correlation analysis was conducted. Figure 6 shows the scatter plots of the naturalness ratings and the “size image” for the proposed and controlled conditions. From the figure, we found a moderate positive correlation (*r* = 0.51, *p* < 0.01) in the proposed condition. This result indicates that the proposed method generates more natural gestures for larger-size words.

### 4.3 Discussion

The above analysis indicates that the proposed condition outperformed the control condition in terms of overall naturalness. Therefore, the results of this study suggest that the proposed method can generate physical images that enhance agency perception.

However, this effect was affected by the word category and the size of the gesture. When dividing the overall effect into the three categories and two sizes, the proposed method outperformed the control method only significantly for the large inanimate category.

Those results partially support the hypotheses presented Section 4.2. The overall main effect of the condition supports the first hypothesis. The interaction between the condition and the categories supports the second hypothesis, suggesting the weak effect of the symbol grounding in an abstract category.

There are several possible reasons why the expected effect was not strongly observed in this experiment. Although we cannot deny the possibility that our assumptions (i.e., artifacts become agents through symbol grounding) are incorrect, more robust results may be obtained by improving experimental settings. For instance, the data obtained in this experiment came from a crowdsourcing survey, which may have introduced noise into the participants’ ratings. We can also consider that mapping the “size image” to the physical image was inadequate. The scatter plots in Figure 6 suggest the latter possibility, indicating that our method does not exhibit sufficiently natural behavior for small-size words. The next experiment explores this possibility.

## 5 Experiment 2: “size image” in shape

This experiment also addresses the first research question by assessing the naturalness of gestures generated from the “size index.” However, to explore the second research question, we employed a different mapping of the “size index” to the body. The method adopted here focuses on the shape modality in Laban theory. From the correlations in Figure 6, it can be speculated that the small movements in Experiment 1 did not appear to be performed as a gesture. Based on this speculation, this experiment examines the research questions by mapping size images to body size so that perceptible gestures are generated even when small-sized words are uttered.

### 5.1 Methods

**FIGURE 7:**
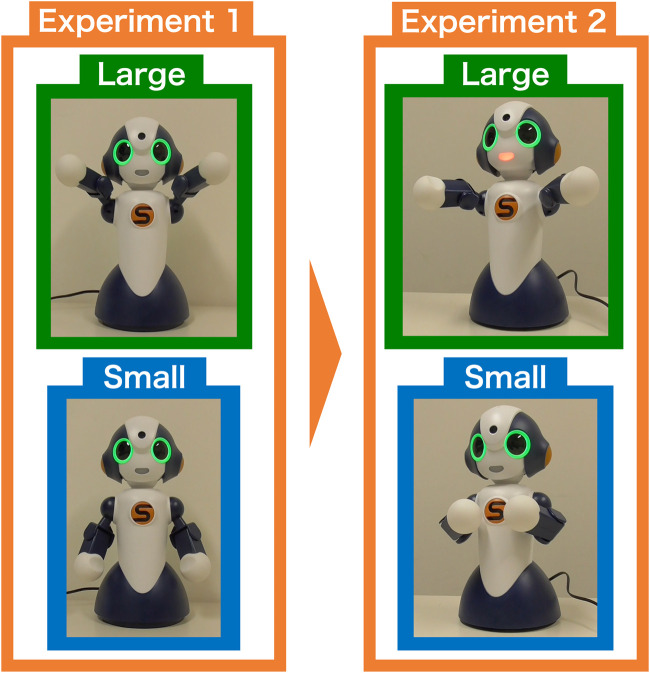
Example of gesture control by posture (top: maximum value, bottom: minimum value).

The experiment method was the same as Experiment 1 except for the movies presented to participants. In the movies in this experiment, we arranged the correspondence between the “size image” and the physical image to express the size of the posture. The default posture of the robot is the same as in Experiment 1. In addition to the body parameters (arms and shoulders) used in the previous experiment, the neck joints were controlled according to the “size image” of the word. Table 4 shows the minimum and maximum values of the word “size image” and the corresponding values of Sota’s joint angle parameters for the proposed and controlled methods. All the pictures showing the finishing pose used in this experiment are presented in the Supplementary Material. Examples from them are shown in Figure 7.

As illustrated in 4.1.1, the upper left and lower left images are gestures for uttering the word with the largest (pyramid) and smallest (tick) “size image” in Experiment 1. In this experiment, we changed these gestures as shown in the upper right and lower right images of Figure 7. When the “size image” was largest, the parameters of the arm and shoulder were set at the position where the distance between the arm and the arm was the largest, and the neck was also set upward. When the “size image” was the smallest, the arm and shoulder parameters were set at the position where the arm-to-arm distance was the smallest, and the neck was also set downwards. As can be seen in Figure 7, the camera position was also changed to make the differences in posture obvious.

### 5.2 Results

**FIGURE 8:**
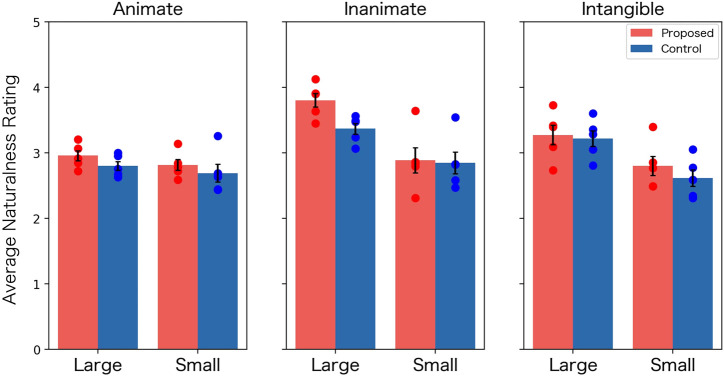
Mean rating of naturalness in Experiment 2 (Error bars: standard errors, Dots: ratings for naturalness for the five words).

As in Experiment 1, 300 participants were recruited from Lancers joined the experiment. 43 participants who incorrectly responded to the dummy questions were excluded from the analysis. From the remaining 257 participants’ responses, the average ratings for 29 words in two conditions were calculated. Using the calculated value as the unit of analysis, the naturalness ratings were summarized in Figure 8.

From the data shown in the figure, we tested the same hypotheses in Experiment 1. We report the results of the statistical tests of the main effect of the condition, and the second-order interaction between the condition, size and categories, and the first-order interaction between the condition and the categories from the three-way [condition (proposed vs. controlled) × size (large vs. small) × category (animate vs. inanimate vs. intangible)] ANOVA performed for Figure 6. As a result, we obtained a significant main effect of the condition (*F* (1, 48) = 4.14, *p* = 0.04, *q* = 0.08, *η* ^2^ = 0.09) and no significant interactions involving the condition (the second-order interaction: *F* (1, 48) = 0.91, *p* = 0.41, *q* = 0.57, *η* ^2^ = 0.04, the interaction between condition and category: *F* (2, 48) = 0.20, *p* = 0.81, *q* = 0.81, *η* ^2^ = 0.22). These results indicate that the difference in naturalness between conditions is not affected by category or size.

**FIGURE 9:**
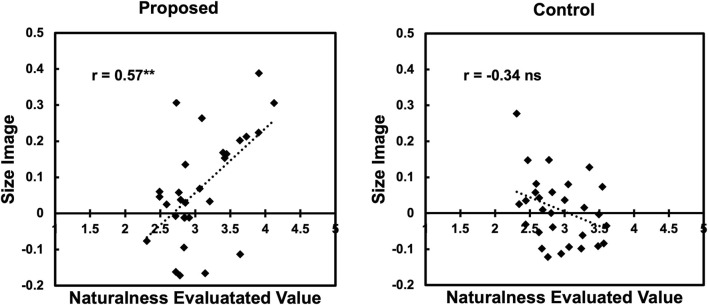
Correlation between naturalness and “size image” in Experiment 2.

As in Experiment 1, we also conducted a correlation analysis. Figure 9 shows the scatter plots of the naturalness scores and the “size image” for the proposed condition. From the figure, we found a moderately positive correlation (*r* = 0.57, *p* < 0.01) in the proposed condition as in Experiment 1. This result indicates that the larger the value of “size image,” the more natural the image tends to be evaluated.

### 5.3 Discussion

In Experiment 2, we reexamined the research questions using a different mapping of the “size image” to the physical image than in Experiment 1. The results showed that, as in Experiment 1, the proposed method produced more natural images than the control method. However, contrary to Experiment 1, Experiment 2 found no interaction between the condition and the other factors. Therefore, we can assume that the gestures generated in Experiment 2 can be applied to more general situations to cause an agency perception.

However, the effect size of the condition obtained in Experiment 2 was smaller than that in Experiment 1. In addition, as in Experiment 1, we observed a correlation between the “size image” and naturalness ratings. This suggests that the small-sized words in Experiment 2 also exhibited unnatural gestures.

## 6 General discussion

This study was guided by two research questions. Concerning the first question (how can “size images” evoking an agency perception be extracted from the vector space of word-distributed representations?), we assessed a method proposed in our previous study (Sasaki et al., 2023) as one potential answer. The observed difference between the proposed and the control conditions indicates the necessity of selecting appropriate synonyms for constructing the “size index.” The current study has demonstrated the advantage of our modification (Sasaki et al., 2023) from the previous method (Grand et al., 2022).

Furthermore, for the second question (what forms of gesture expression are effective in constructing natural interaction based on the agency perception?), by comparing the results of the two experiments, we can assume that expression using the mapping in the posture has a more general effect. However, in common with both experiments, there was a positive correlation between the naturalness rating and the “size image.”

The fact that the gestures corresponding to smaller “size images” did not receive good ratings requires further examination. In the discussion of Experiment 1, it was considered that the small amount of movement was a factor causing the low naturalness ratings. However, neither the small “size image” nor a large amount of movement in Experiment 2 improved the naturalness rating for small-sized words. These results may suggest an asymmetry between the small and large poles of the “size index”; Words at the smallest pole may be less associated with the body, while words at the largest pole may be more associated with the body.

We also need to consider the influence of categories on the effectiveness of the proposed method. Previous studies have noted that abstract concepts are less grounded in the physical experience (Lakoff and Johnson, 1980; Utsumi, 2020). Consistent with this discussion, in Experiment 1, only one of the concrete categories demonstrated the effectiveness of the proposed condition. Additionally, the observation that the same word “Mind” appeared in both “large” and “small” abstract concepts indicates the limited extent of symbol grounding.

Our experiment also suggests that using a single size index across various categories has limitations. In this study, we applied the same “size index,” derived from the pairs of synsets listed in Table 2, to all three categories. We did not differentiate categories when calculating the size index because of enhancing the index’s applicability. Considering that the method might be applied to any arbitrary words, it appears difficult to determine the abstractness of a word beforehand. However, our results, particularly the observed differences in the effects of the condition between animate and inanimate categories, clearly indicate the necessity of adjusting the scale of the mapping from “size image” to “physical image.”

## 7 Conclusion

In this study, we started from the hypothesis that symbol grounding is important in generating the agency perception. In line with this hypothesis, we composed a “size image” of a symbol grounded in a quantitative vector space of word-distributed representations. We also explored the hypothesis by examining two mapping of “size image” to body images. The experiments verified the proposed method although the effect size was not large.

We consider that the reason for the small effect size is partially attributed to our approach. Unlike recent research on gesture generation based on deep learning technologies, this research has many assumptions. We especially composed an abstract axis that mediates speech and body. Although these top-down approaches do not reach bottom-up approaches in terms of performance, it is useful to guide the novel interaction design with agents. Thus, we believe our study contributes to theoretical and practical developments in HAI research.

There are several other limitations to this study. The first concerns the gestures with small-sized words as already noted. Even though the problem noted in Section 6 may exist, it is beneficial to invent expressions of small-sized gestures that humans can evaluate as natural. To overcome this problem, we need to improve expression ability in the used body image. The currently used robot (Sota) has limitations in performing detailed gestures. Therefore, to confirm our hypothesis, it may be necessary to use other robots or virtual agents.

The robustness of the results also needs to be improved. The data collection in this study was conducted using crowdsourcing, and a lot of noise was possibly introduced in the data collection process. A future study employing face-to-face situations in a laboratory has the possibility of leading to more insights with the additional effect of the presence of embodied robots.

The model update on the distributed representation might also improve the result of the study. The recent rapid development of natural language processing provides a more naturalistic correspondence between discrete symbols and quantitative images. Although existing LLM hold a problem of explainability, it is useful to include those in our approach for demonstration purposes.

In addition to addressing the issues mentioned above, we plan to explore the physicalization of body images using various indices such as “sharpness” and “fastness,” alongside “size” in future work. We are considering the possibility that such semantic axes could be associated with the dimensions of body movement (space, weight, and time) as proposed in the dance theory (Laban and Ullmann, 1971). This direction for future research aims to bring our method closer to the generation of human gestures. Theories of human gesture (McNeill, 1992) indicate that human gestures encompass many aspects beyond those addressed in this study. Our approach is an endeavor to deconstruct such complex gestures based on the fundamental physical experience (symbol grounding), drawing on several cognitive science theories (Pinker, 2007; Lakoff and Johnson, 1980; Tversky, 2019; Hawkins, 2021). We believe that this foundational research will ultimately contribute to the development of advanced artifacts capable of seamless interaction with humans, featuring a mechanism for converting between human symbols and quantitative representations.

## Data Availability

The raw data supporting the conclusion of this article will be made available by the authors, without undue reservation.

## Footnotes

1 The distance between the words “large” and “small” is quite close within the entire space of word-distributed representation because both are commonly used in contexts describing the size of an object. Therefore, poles defined solely by these two words have limited distinguishability for other words in terms of size.

2 We can find more than sixty citations in Google Scholar at the point of submission.

3
https://www.vstone.co.jp/english/index.html

4 This duration corresponds the time to change the pose from the default to the final position. Aside from this time, the proposed method also requires time to load the model (JWikiEntVec) and compute the size image. The average of the initial loading time is 6.28 s (*n* = 10), which is required only once to initialize the entire system. After the initial setup, the method only requires 0.59 s, on average (*n* = 10), to compute the size images. Those computational times were recorded by a 2020 MacBook Pro with an Apple M1 CPU.

5 These words were assumed to be samples of *Large*_*h*_ and *Small*_*h*_ for each category. There might be several confounding factors, among which we examined iconicity rating as one of the factors possibly influencing gesture perception. However, from the publicly available dataset of iconicity ratings (Thompson et al., 2020; Winter et al., 2023), we could not find clear evidence of the influence on gesture perception in our experiment. Supplementary Material show the details of this analysis.

6 The movement of the shoulder and arm at the minimum value is generated by taking the parameter of the smallest unit of movement of the internal motor.

7 The average rating for “Mind” was included both for large intangible and small intangible conditions.
