# Supplementary material for: Evaluation of co-speech gestures grounded in word-distributed representation
Source: Front Robot AI. 2024 Apr 25;11:1362463. doi: 10.3389/frobt.2024.1362463 (PMC11079185; doi:10.3389/frobt.2024.1362463)
Supplement: Supplementary file 3 [file DataSheet3.docx]

Iconicity of *Large_h_* and *Small_h_*

Iconicity is a resemblance between form and meaning grounded in perceptuo-motor analogy" (Thompson et al., 2020). It is also stated that "Iconic words and signs are characterized by a perceived resemblance between aspects of their form and aspects of their meaning" (Winter et al., 2023). The “forms” noted here differ between types of languages. In verbal languages, phonological elements (i.e., syllables) construct the form of words, while sign languages have a distinct form of signs constructed by body posture and movements.

From the above definition of iconicity, this concept has a possibility to influence the perception of generated gesture. Concerning the publicly available data on iconicity rating, we found several ones for verbal language but not for sign language and gestures. Therefore, we investigated the availability of the rating scores for our experimental materials in those verbal language data. We first accessed a dataset collecting iconicity ratings for Japanese words (Thompson, et al., 2020). However, we found that only two words, Dream (yu-me) and Mind (ko-ko-ro), appeared in the Japanese dataset.

We also check the score by using the English forms for the corresponding meaning of the words in the larger dataset (Winter et al., 2023). We found that 21 out of 29 words had scores in the dataset and summarized the result in the table of this supplementary material. The words not included in the dataset are as follows: Daphnia (mi-ji-n-ko), tick (da-ni), Tokyo Sky Tree (to-kyo-su-ka-i-tu-ri), Mt Fuji (fu-ji-sa-n), Tokyo Tower (to-kyo-to-wa), Pyramid (pi-ra-mi-ddo), and microtip (ma-i-ku-ro-chi-ppu). This suggests a biased distribution, as many large inanimate concepts are absent from the dataset, primarily due to indicating names of concrete artifacts. The familiarity of those words is heavily influenced by cultural factors. Consequently, we encountered difficulty in using rating scores to control the materials for the experiments in this study.

Animate

| Large | | Small | |
| --- | --- | --- | --- |
| Word | Iconicity rating | Word | Iconicity Rating |
| Elephant (zo-u) | 4.4 | Ant (a-ri) | 4.36363636 |
| Whale (ku-ji-ra) | 3.9 | Daphnia (mi-ji-n-ko) |  |
| Giraffe (ki-ri-n) | 3.5833333 | Mosquito (ka) | 2.8 |
| Bear (ku-ma) | 3.90909091 | Tick (da-ni) | 5.54545455 |
| Hippopotamus (ka-ba) | 2.6 | Fleas (no-mi) |  |
| Mean | 3.67848484 |  | 4.23636364 |

Inanimate

| Large | | Small | |
| --- | --- | --- | --- |
| Word | Iconicity rating | Word | Iconicity Rating |
| Tokyo Sky Tree  (to-kyo-su-ka-i-tu-ri) |  | Sand (su-na) | 3 |
| Mt. Fuji (fu-ji-sa-n) |  | Beads (bi-zu) |  |
| Tokyo Tower (to-kyo-ta-wa) |  | Needle (ha-ri) | 3.7 |
| Everest (e-be-re-su-to) |  | Microchip (ma-i-ku-ro-chi-ppu) |  |
| Pyramid (pi-ra-mi-ddo) | 3.1 | Screw (ne-ji) | 5.58333333 |
| Mean | 3.67848484 |  | 4.23636364 |

Animate

| Large | | Small | |
| --- | --- | --- | --- |
| Word | Iconicity rating | Word | Iconicity Rating |
| Elephant (zo-u) | 4.4 | Ant (a-ri) | 4.36363636 |
| Whale (ku-ji-ra) | 3.9 | Daphnia (mi-ji-n-ko) |  |
| Giraffe (ki-ri-n) | 3.5833333 | Mosquito (ka) | 2.8 |
| Bear (ku-ma) | 3.90909091 | Tick (da-ni) | 5.54545455 |
| Hippopotamus (ka-ba) | 2.6 | Fleas (no-mi) |  |
| Mean | 3.67848484 |  | 4.23636364 |
