# Supplementary material for: Evaluation of co-speech gestures grounded in word-distributed representation
Source: Front Robot AI. 2024 Apr 25;11:1362463. doi: 10.3389/frobt.2024.1362463 (PMC11079185; doi:10.3389/frobt.2024.1362463)
Supplement: Supplementary file 8 [file DataSheet2.docx]

**Video Material Experiment 2**

**Top Synset**

**Animate (Large)**

**
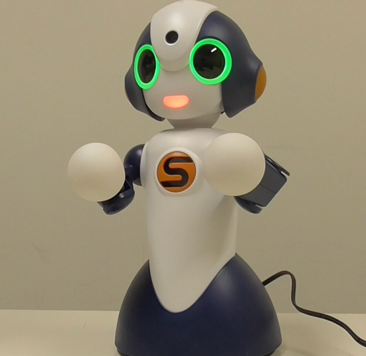

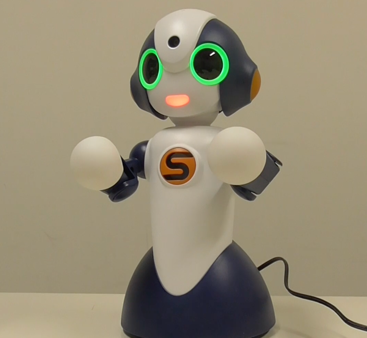

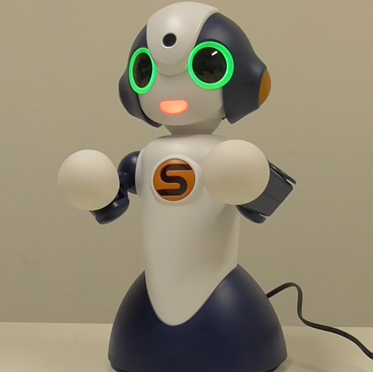
**

**Elephant Whale Giraffe**

**
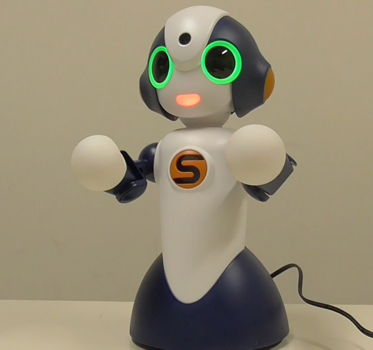

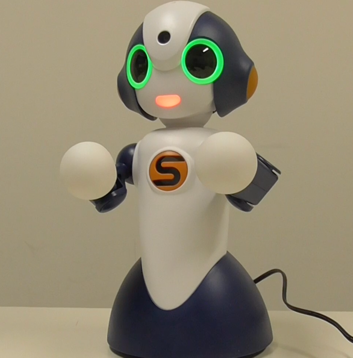
**

**Bear Hippopotamus**

**Animate (Small)**

**
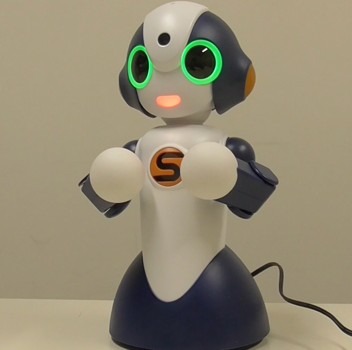

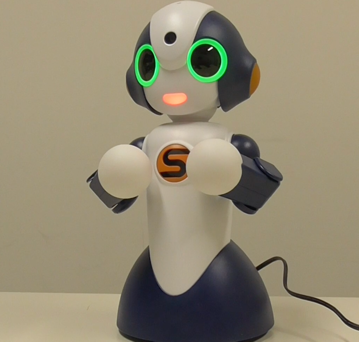

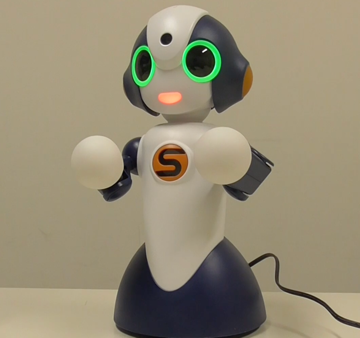
**

**Ant Daphnia Mosquito**

**
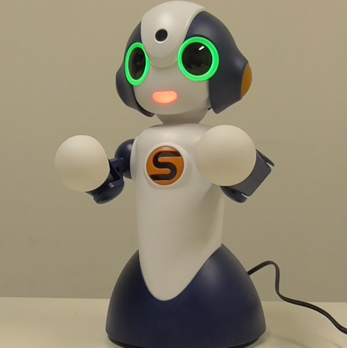

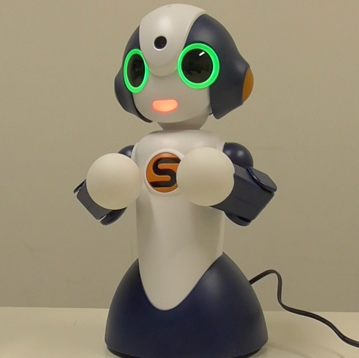
**

**Tick Fleas**

**Inanimate (Large)**

**
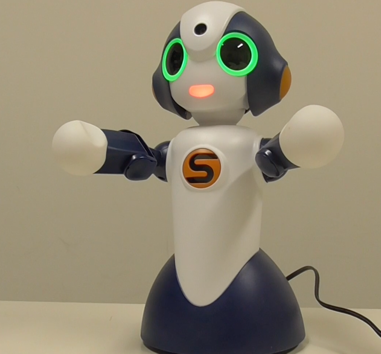

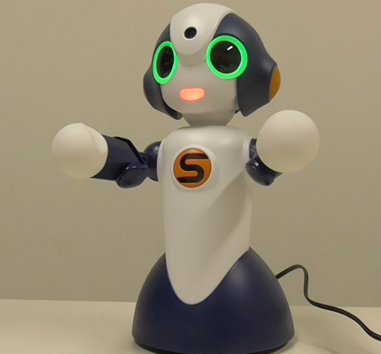

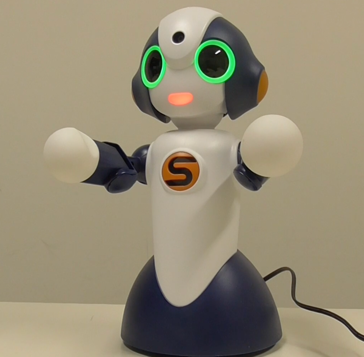
**

**
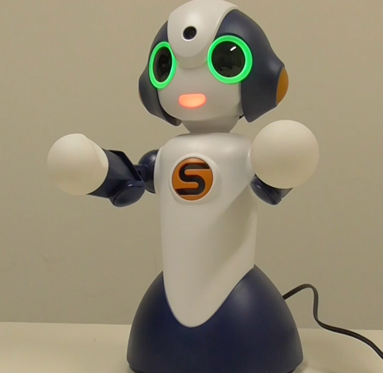
**

**Tokyo Sky Tree Mt. Fuji Tokyo Tower**

**
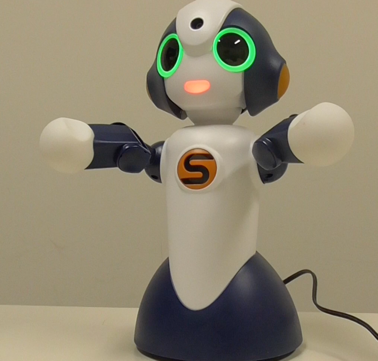
**

**Everest Pyramid**

**Inanimate (Small)**

**
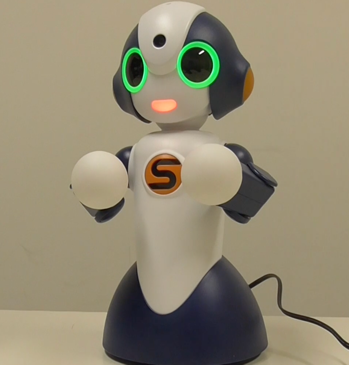

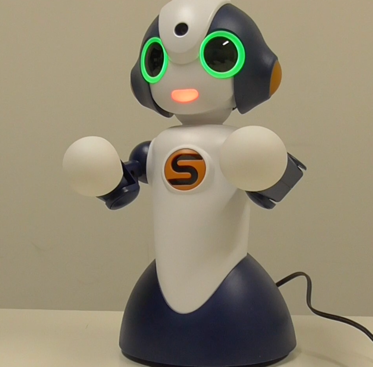

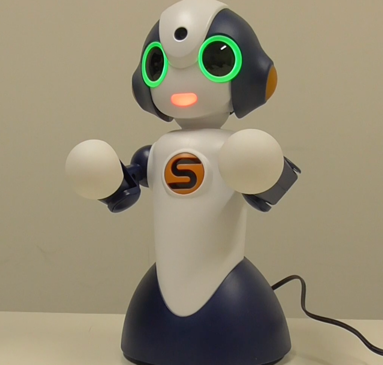
**

**Sand Beads Needle**

**
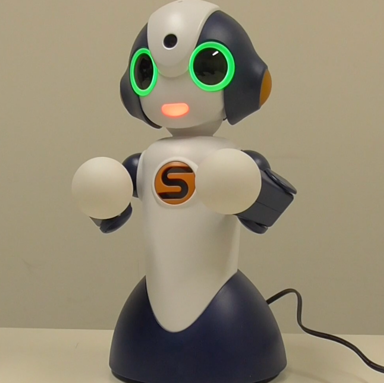
**

**
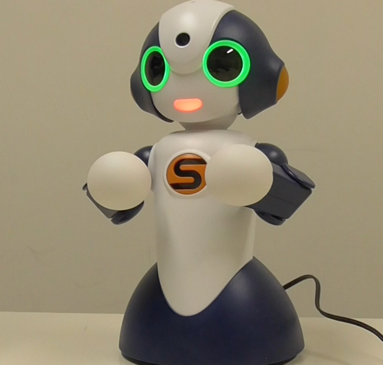
**

**Microchip Screw**

**
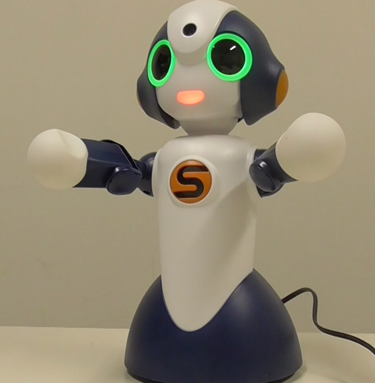

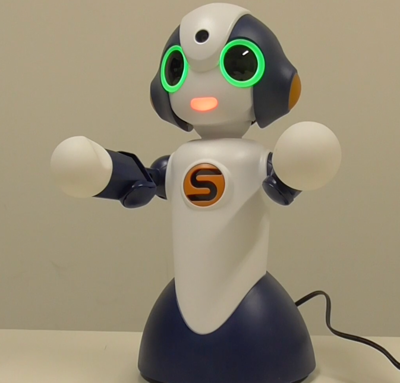

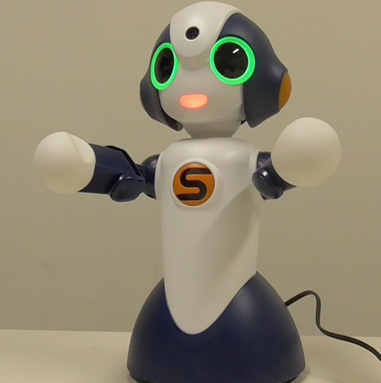
Intangible (Large)**

**Space Love Dream**

**
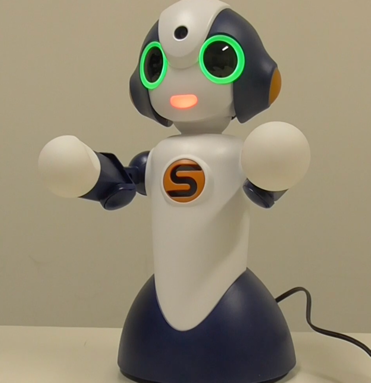

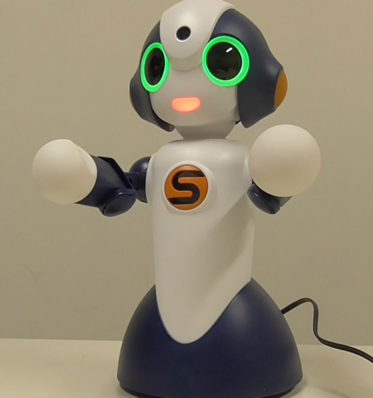
**

**Mind Sea**

**
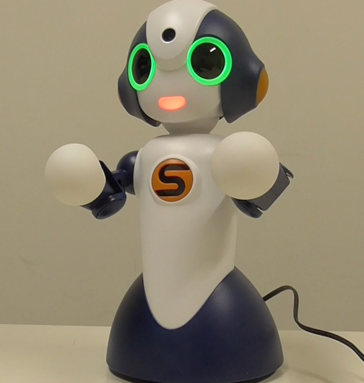

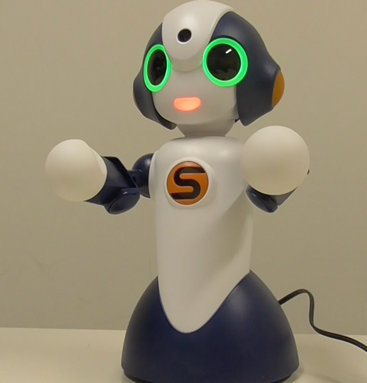

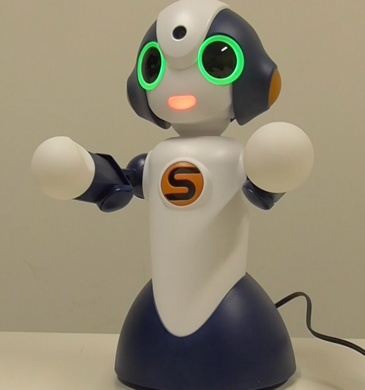
Intangible (Small)**

**Mind Jealousy Envy**

**
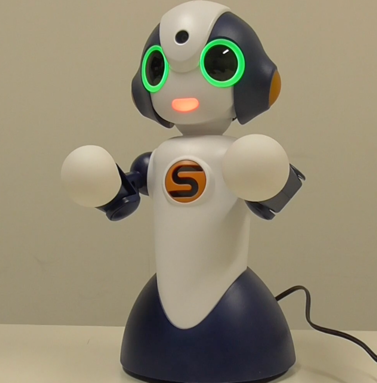

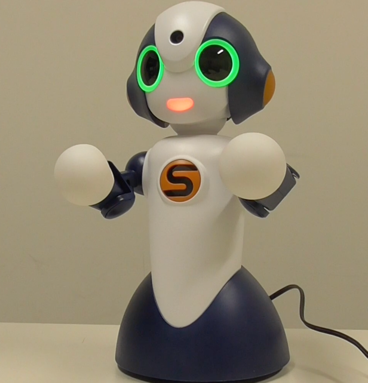
**

**Vanity Point**

**Bottom Synset**

**
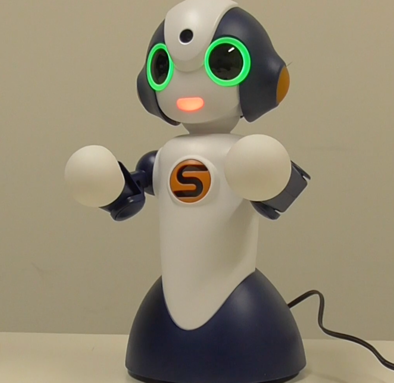

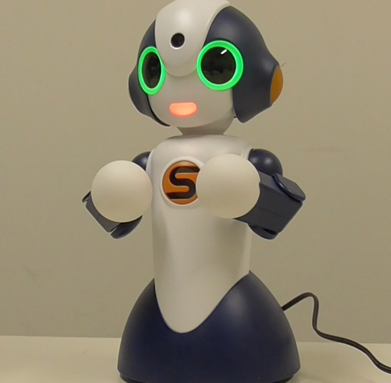
Animate (Large)**

**
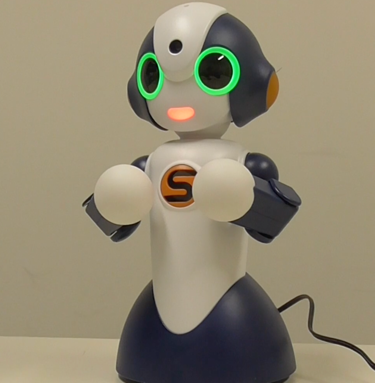
**

**Elephant Whale Giraffe**

**
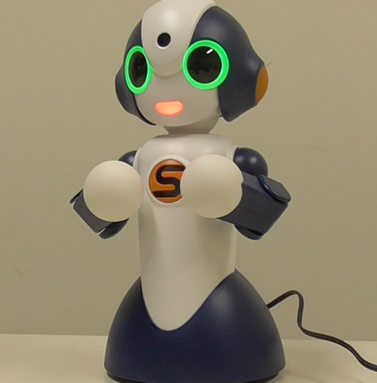

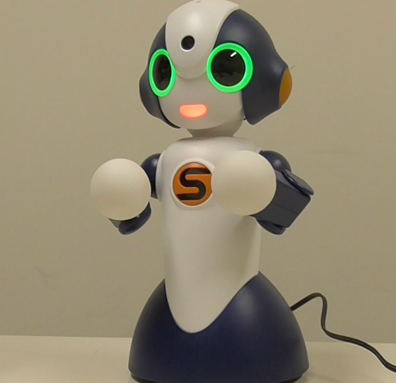
**

**Bear Hippopotamus**

**
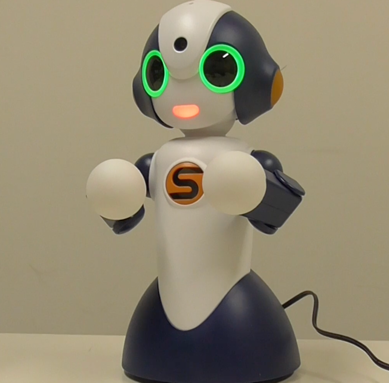

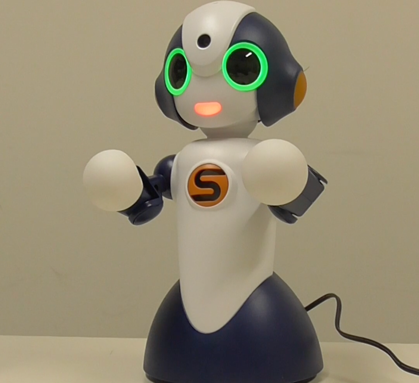
Animate (Small)**

**
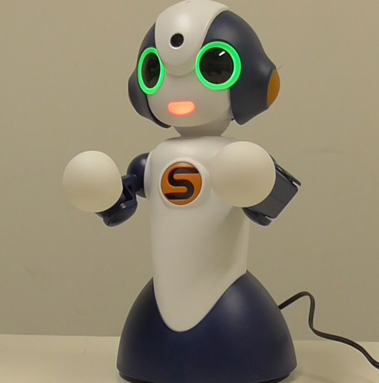
**

**Ant Daphnia Mosquito**

**
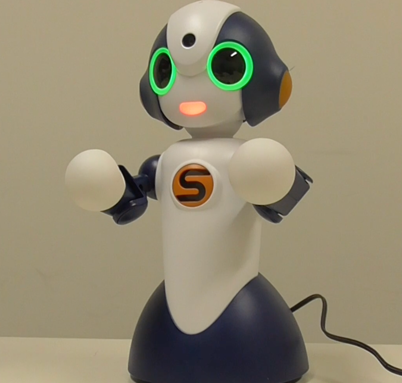

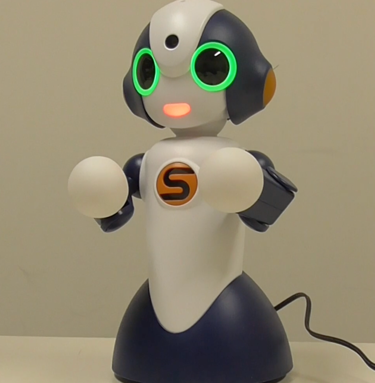
**

**Tick Fleas**

**
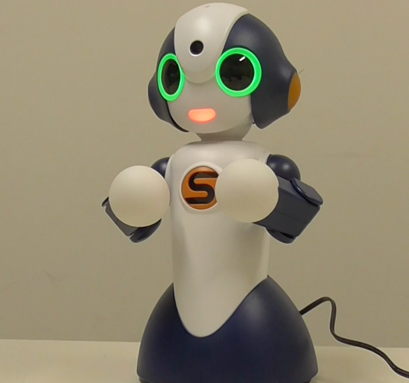
Inanimate (Large)**

**
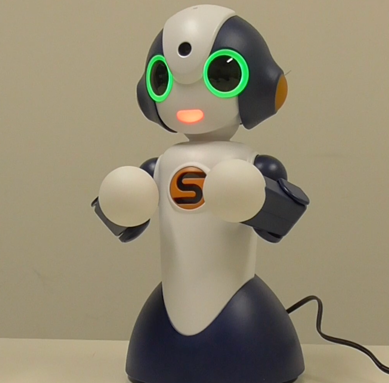

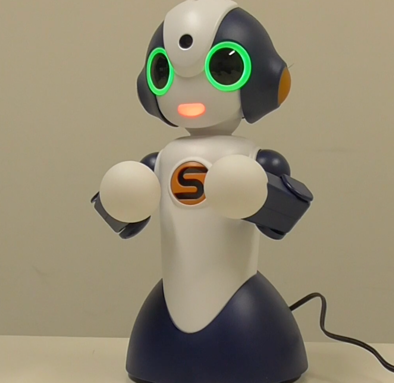
**

**Tokyo Sky Tree Mt. Fuji Tokyo Tower**

**
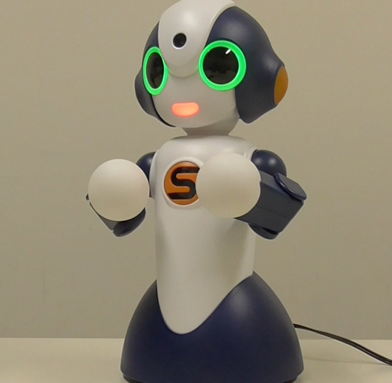

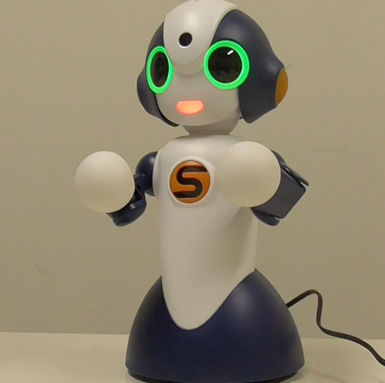
**

**Everest Pyramid**

**
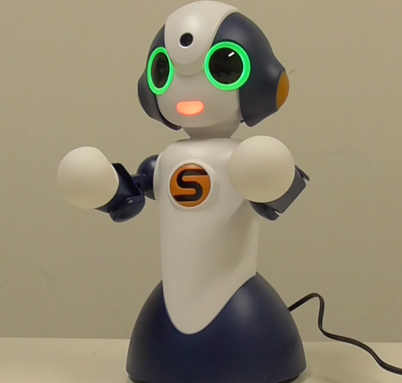

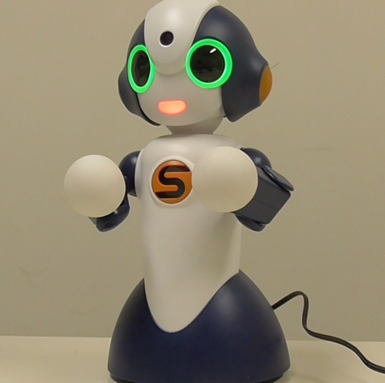

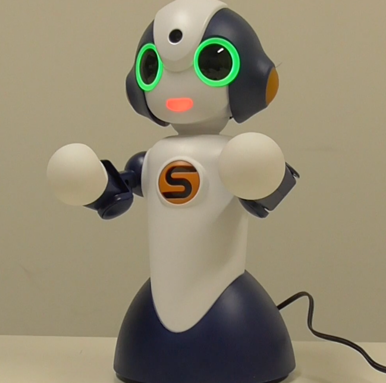
Inanimate (Small)**

**Sand Beads Needle**

**
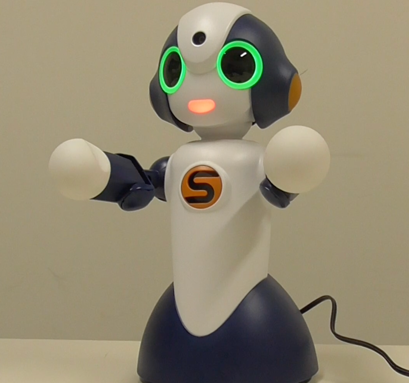

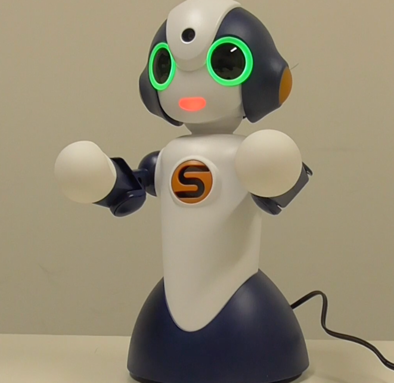
**

**Microchip Screw**

**
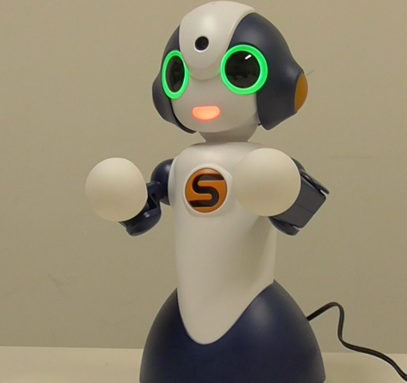

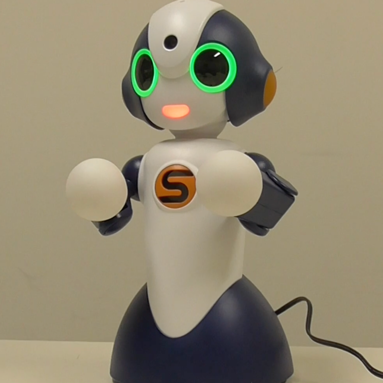

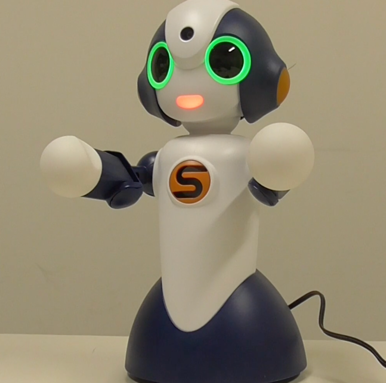
Intangible (Large)**

**Space Love Dream**

**
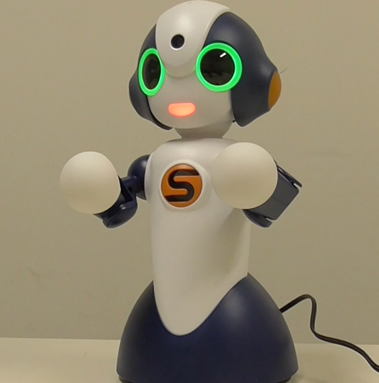

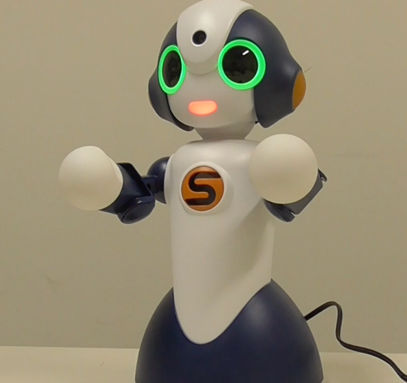
**

**Microchip Screw**

**
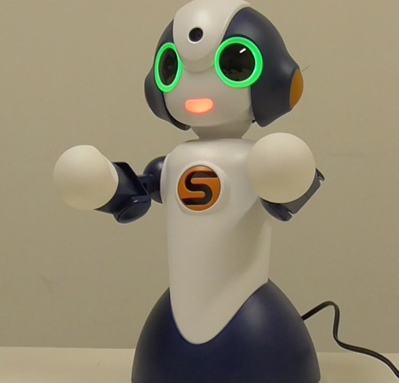

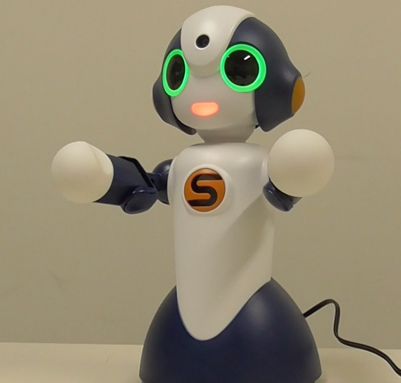
Intangible (Small)**

**
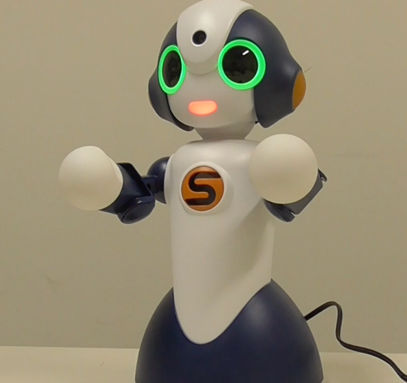
**

**Mind Jealousy Envy
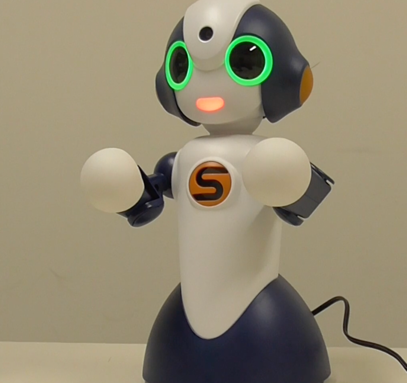

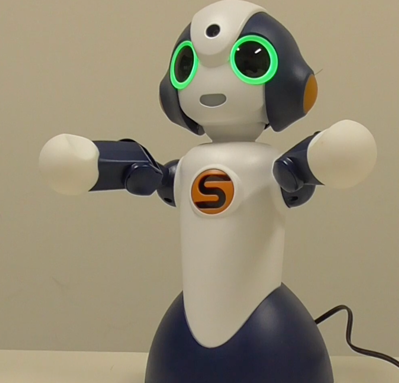
**

**Vanity Point**
