# Supplementary material for: Evaluation of co-speech gestures grounded in word-distributed representation
Source: Front Robot AI. 2024 Apr 25;11:1362463. doi: 10.3389/frobt.2024.1362463 (PMC11079185; doi:10.3389/frobt.2024.1362463)
Supplement: Supplementary file 15 [file DataSheet1.docx]

**Video Material Experiment 1**

**Top Synset**

**Animate (Large)**


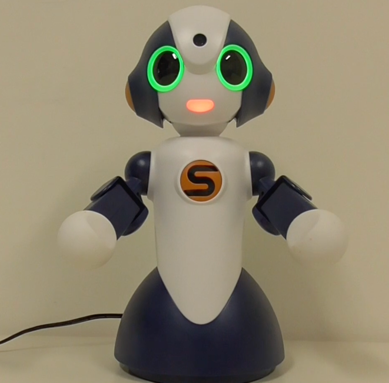

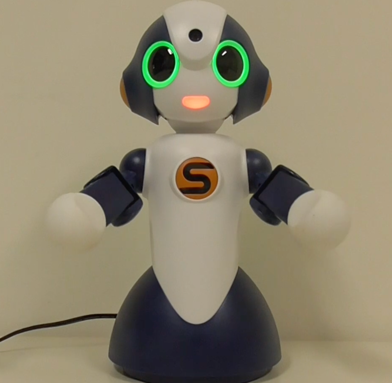

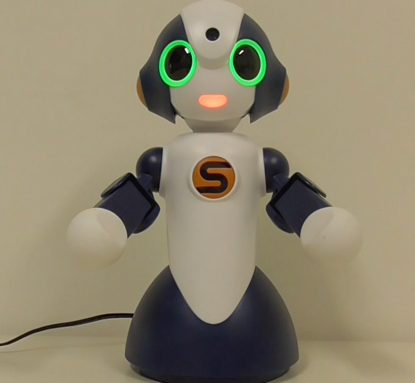


**Elephant Whale Giraffe**


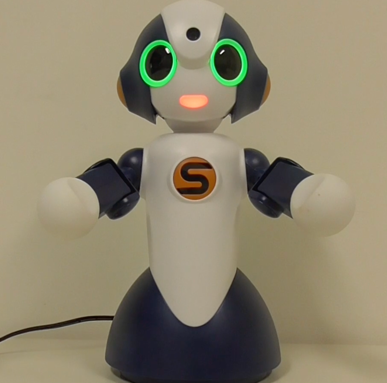

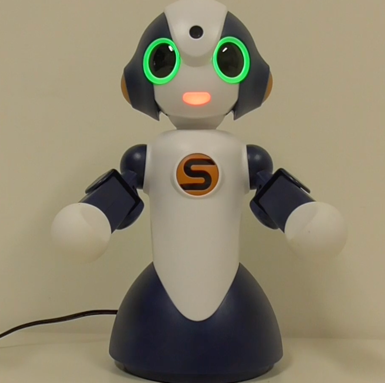


**Bear Hippopotamus**


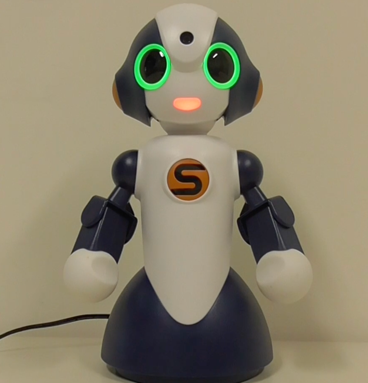

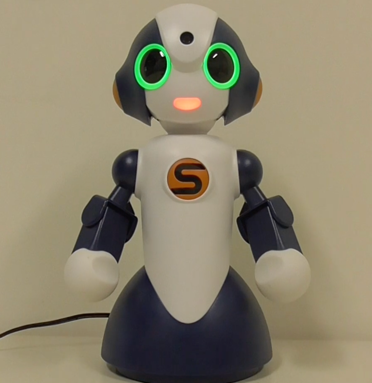
**Animate (Small)**


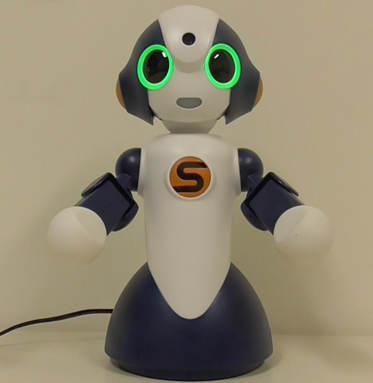


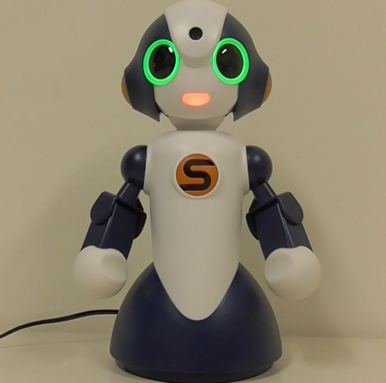
 **Ant Daphnia Mosquito**


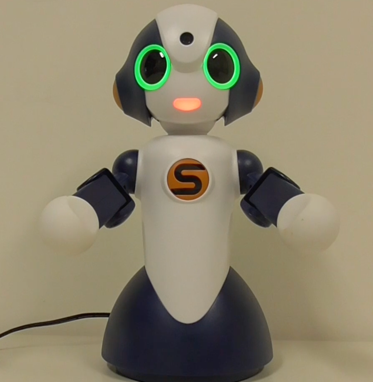


**Tick Fleas**

**Inanimate (Large)**


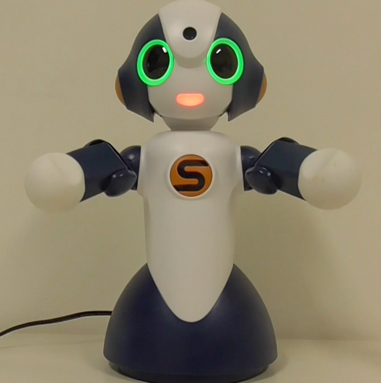

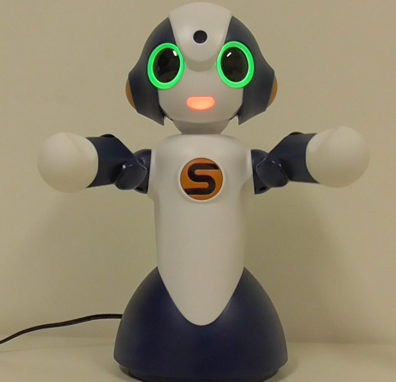

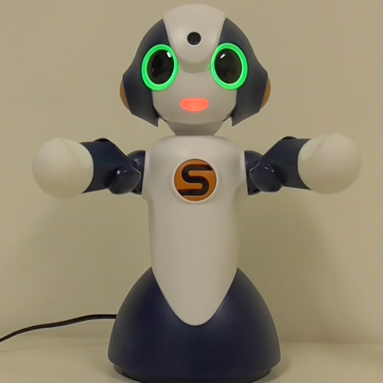


**Tokyo Sky Tree Mt. Fuji Tokyo Tower**


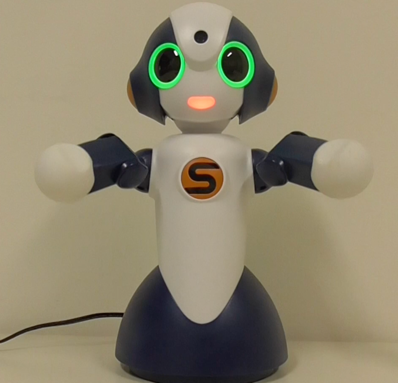

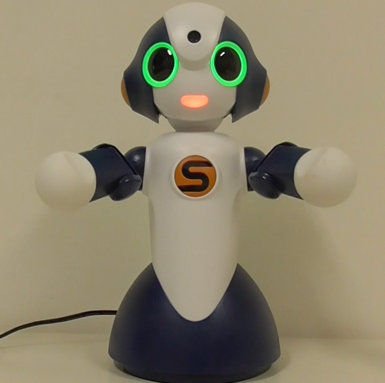


**Everest Pyramid**

**Inanimate (Small)**


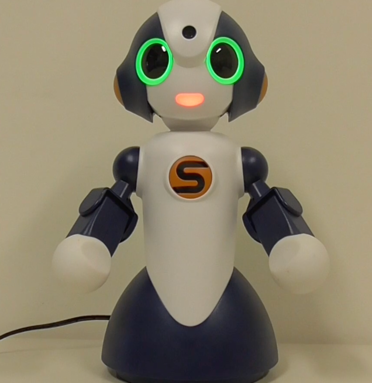

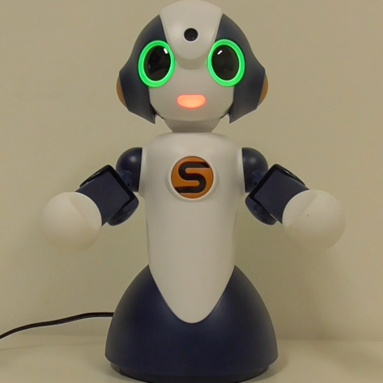

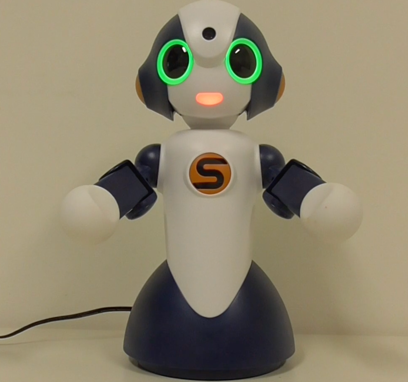


**Sand Beads Needle**


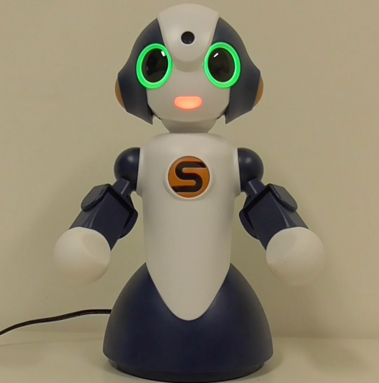

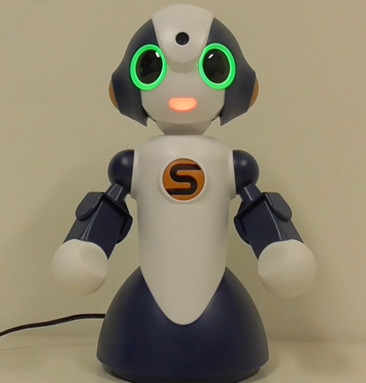


**Microchip Screw**


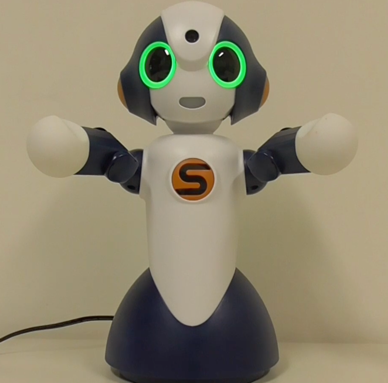

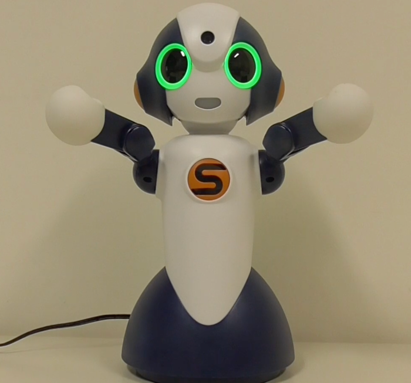
**Intangible (Large)**


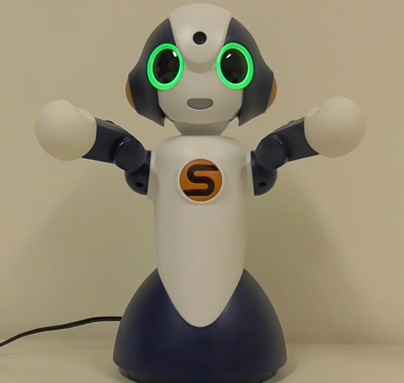


**Space Love Dream**


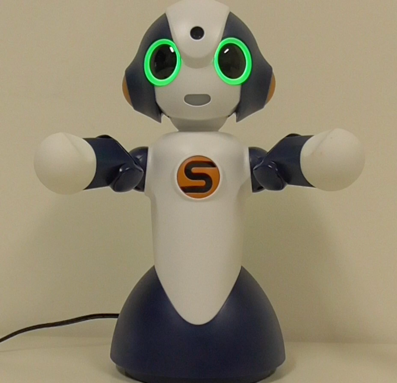

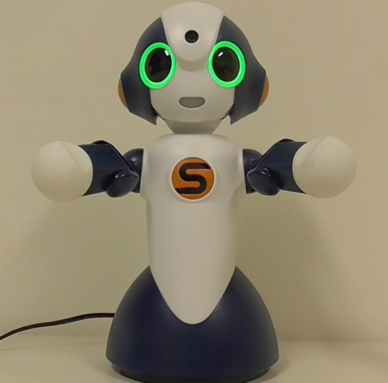


**Mind Sea**


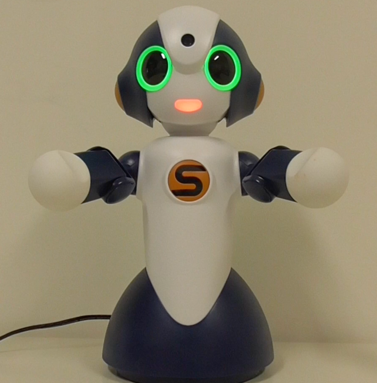

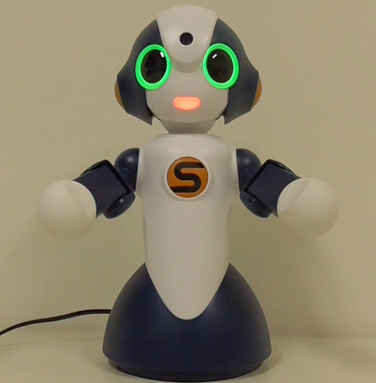
**Intangible (Small)**


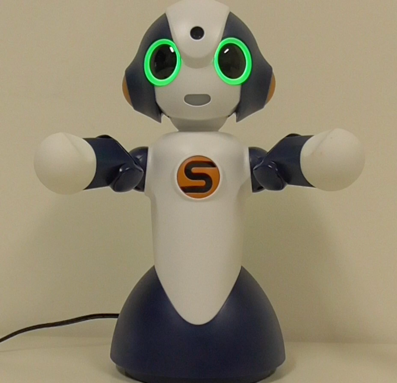


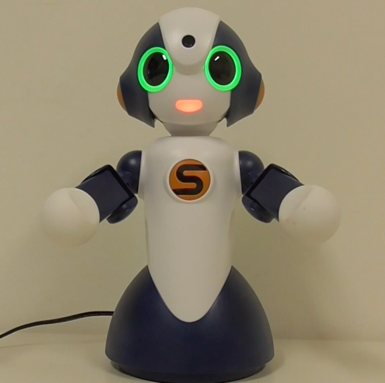

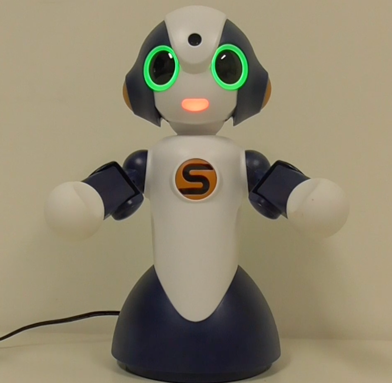
 **Mind Jealousy Envy**

**Vanity Point**

**Bottom Synset**

**Animate (Large)**


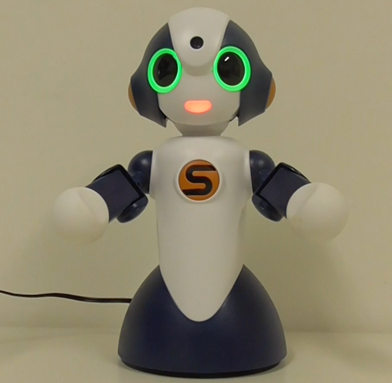

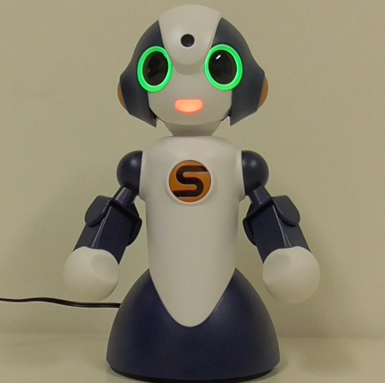

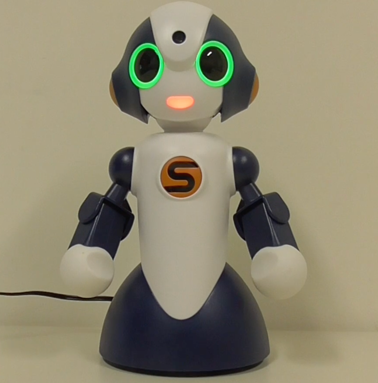


**Elephant Whale Giraffe**


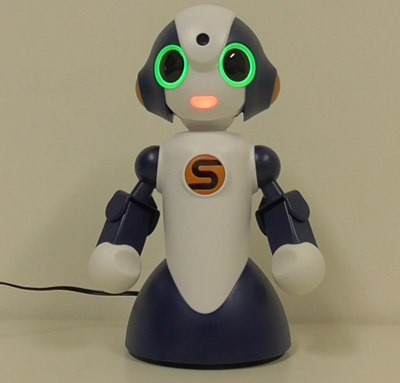

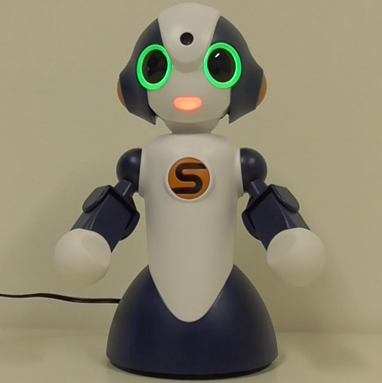


**Bear Hippopotamus**


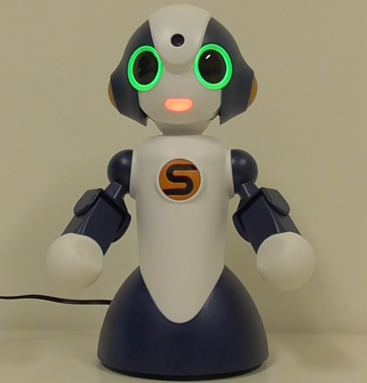

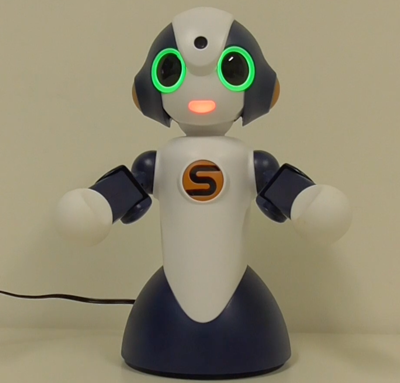
**Animate (Small)**

**
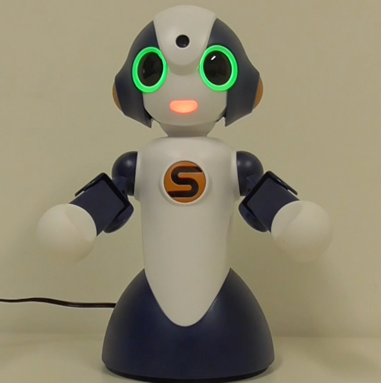
**

**Ant Daphnia Mosquito**


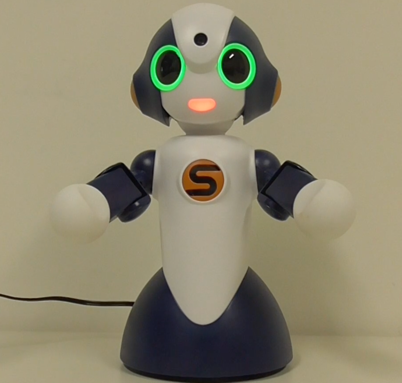

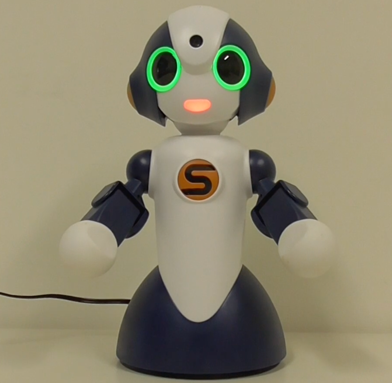


**Tick Fleas**

**
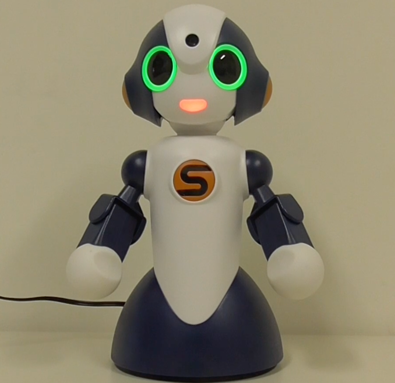

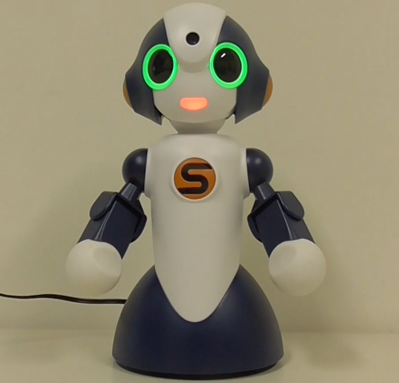
Inanimate (Large)**

**
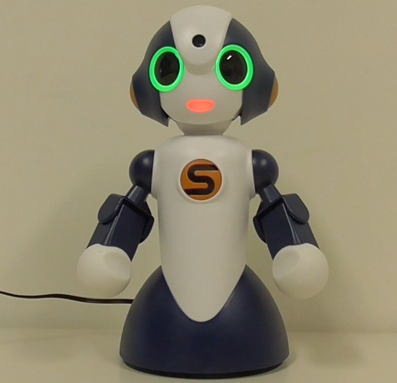
**

**
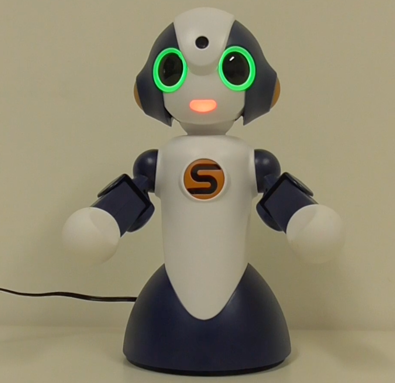

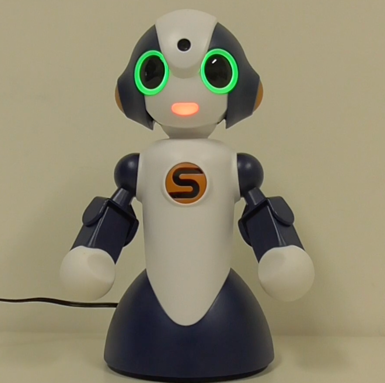
 Tokyo Sky Tree Mt. Fuji Tokyo Tower**

**Everest Pyramid**

**Inanimate (Small)**


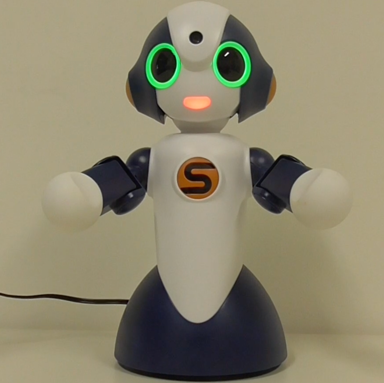

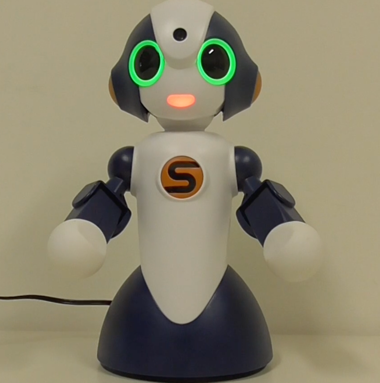

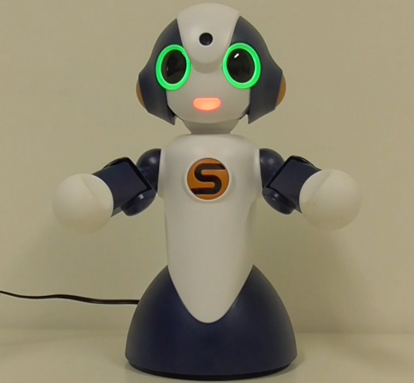


**Sand Beads Needle**


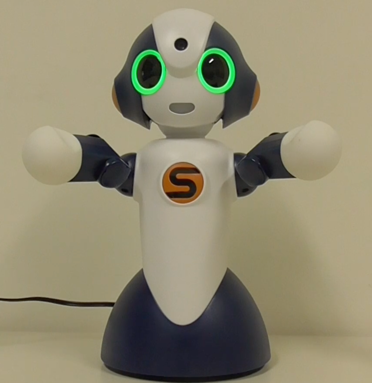

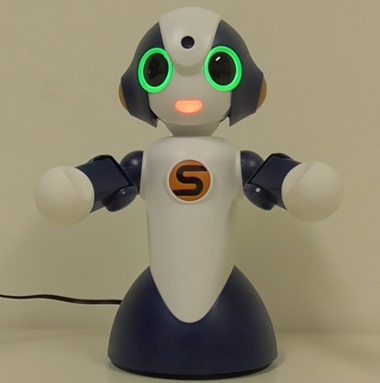


**Microchip Screw**

**Intangible (Large)**


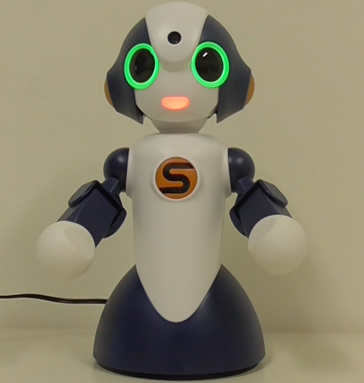

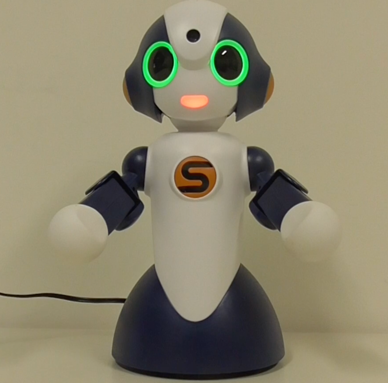

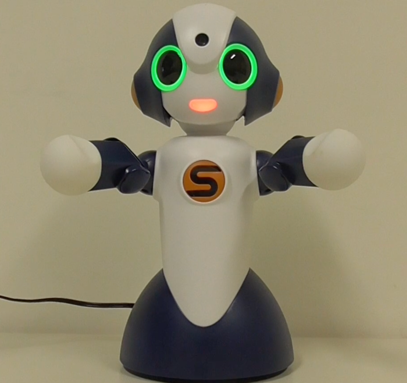


**Space Love Dream**


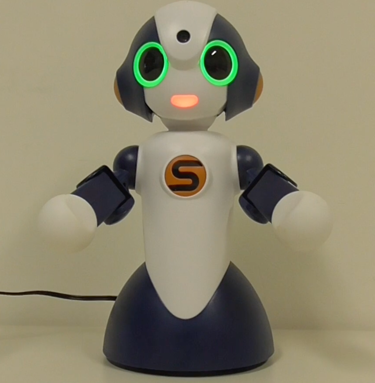

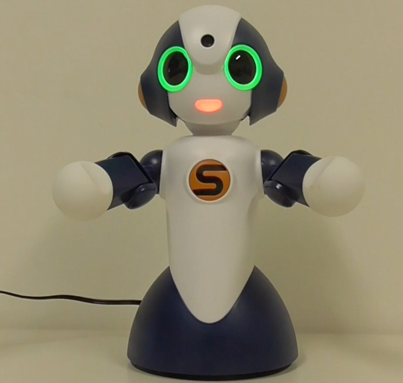


**Mind Sea**

**Intangible (Small)**

**
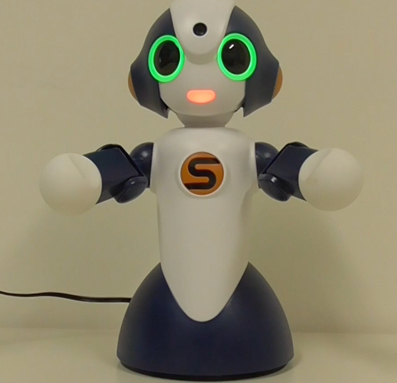

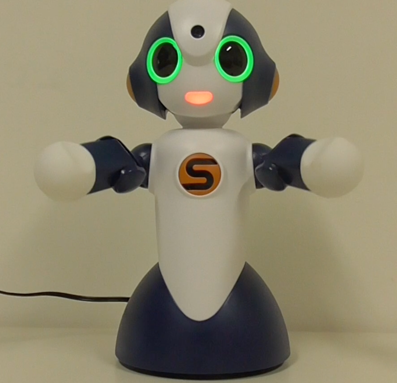
**
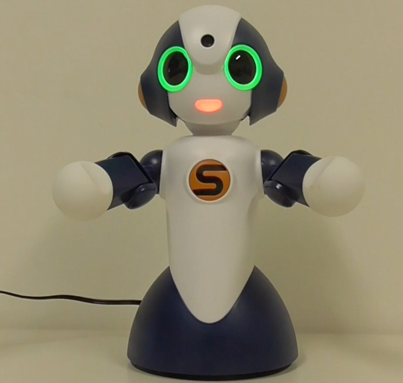


**Mind Jealousy Envy**

**
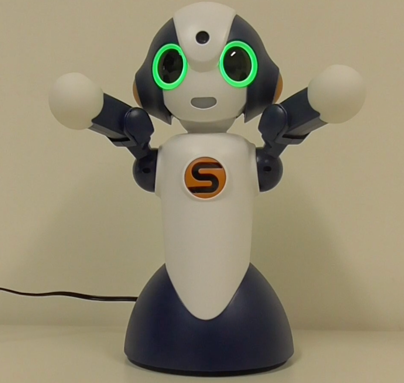

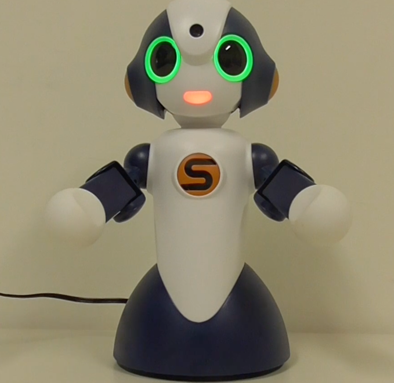
**

**Vanity Point**
